# Prevalence and genetic characterization of methicillin-resistant *Staphylococcus aureus* in Commercial aquaculture farms in Egypt

**DOI:** 10.1038/s41598-026-40144-y

**Published:** 2026-04-10

**Authors:** Maged El-Ashker, Stefan Monecke, Mayada Gwida, Maha Rezk, Elke Müller, Thoraya Saad, Paul Akinduti, Ralf Ehricht

**Affiliations:** 1 Department of Internal Medicine and Infectious Diseases, Faculty of Veterinary Medicine, Mansoura University, Mansoura, 35516 Egypt; 2 Leibniz Institute of Photonic Technology, Member of the Research Alliance “Leibniz Health Technologies” and the Leibniz Centre for Photonics in Infection Research (LPI), 07745 Jena, Germany; 3 InfectoGnostics Research Campus, Centre for Applied Research, Jena, Germany; 4 Center for Translational Medicine (CETRAMED), Jena University Hospital, Friedrich Schiller University Jena, 07747 Jena, Germany; 5 Department of Hygiene and Zoonoses, Faculty of Veterinary Medicine, Mansoura University, Mansoura, 35516 Egypt; 6 Laboratory of Fish Diseases, Research and Management Unit, Damietta Branch, Animal Health Research Institute, Agriculture Research Centre, Giza, Egypt; 7 Department of Medical Laboratory Science, Babcock University, Ilishan, Nigeria; 8 Institute of Physical Chemistry, Friedrich Schiller University, Jena, Germany

**Keywords:** Aquaculture, *Staphylococcus aureus*, Antimicrobial resistance, DNA-microarray, MALDI-ToF, Lateral flow assay, MRSA, Virulence determinants, Genetics, Microbiology, Antimicrobials, Bacteria, Microbial genetics

## Abstract

Methicillin-resistant *Staphylococcus aureus* (MRSA) is a major public health concern due to its resistance and pathogenic potential. This study investigated the prevalence and genetic characterization of MRSA and methicillin-sensitive *S. aureus* (MSSA) in aquaculture farms in Damietta Governorate, Egypt. Sampling was conducted across three commercial farms cultivating shrimp, marine fish, and freshwater fish. A total of 509 specimens were collected, including 150 shrimp, 75 each of sea bass, sea bream, catfish, and tilapia, 15 farm water samples, and 44 samples from farm workers. *S. aureus* was isolated using classical techniques and characterized by MALDI-TOF MS, a PbP2a lateral flow assay, and DNA microarrays. Sixty isolates (11.8%) were identified, comprising 46 MRSA and 14 MSSA strains. The carriage rates of *S. aureus* and MRSA were 6.7% and 4.7% in shrimp, 11.3% and 8.6% in marine fish, 8% and 6.6% in freshwater fish, respectively and were notably higher among farm workers (71.5% and 42.9% in shrimp farms; 50% and 42.9% in marine farms). Four MRSA clonal complexes (CC88, CC361, CC15, CC152) and two MSSA complexes (CC1, CC361) were identified. MRSA CC15 was restricted to shrimp and freshwater fish, while CC152 was found only in workers. Several MRSA clones exhibited multidrug resistance and carried virulence genes encoding leukocidins, hemolysins, proteases, and adhesion & biofilm factors. This is the first report of these clonal complexes in Egyptian aquaculture. The presence of MRSA clones in aquatic ecosystems could represent a significant route for the transmission and spread of multiple pathogenic factors to human populations and emphasize the need to expand monitoring of antimicrobial resistance (AMR) in the study regions.

**Supplementary Information:**

The online version contains supplementary material available at 10.1038/s41598-026-40144-y.

## Introduction

Aquaculture is one of the fastest-growing agricultural sectors, providing a crucial protein source for humans^1^. Global fish production exceeded 178 million tons in 2020, with aquaculture contributing nearly half (87.5 million tons). About 88% of this yield is consumed by 3.2 billion people, meeting 17% of global animal protein needs and supporting food security^2^. In Egypt, aquaculture expanded from 24,000 tons (15.4% of national production) in 1982 to 1,576,189 tons (78.7%) by 2021, driven by hatchery development^3^. Yet limited hatchery capacity, especially for marine species, remains a critical challenge^4^.

Aquatic ecosystems are considered hotspots for resistance and virulence gene exchange^5^. In aquaculture, microbial communities exposed to nutrients, antibiotics, and disinfectants promote resistance even at sub-lethal concentrations^6^. Furthermore, recent evidence has demonstrated that horizontal gene transfer between human pathogens and environmental bacteria could potentially link the resistomes of human microorganisms with those of native aquatic pathogens, thus complicating the clinical manifestations and management of infections caused by both^1^.

Fish and shellfish, though nutritionally valuable, are often associated with foodborne pathogens, particularly staphylococci^7–9^. *Staphylococcus aureus* (*S. aureus*) is highly adaptable, notorious for antimicrobial resistance^10^, and capable of surviving in salty environments through biofilm formation^11,12^. This bacterium causes diverse infections, toxin-mediated diseases which can be due to food contamination^7,13,14^. Its enterotoxins, resistant to heat, can trigger rapid food poisoning symptoms^15^. Nearly all strains secrete enzymes and cytotoxins, with many producing exotoxins such as TSST-1, enterotoxins, exfoliative toxins, and leukocidins^16,17^. Over twenty enterotoxin genes have been identified, though many remain poorly characterized^18^.

Methicillin-resistant *S. aureus* (MRSA) is a major concern due to Staphylococcal Cassette Chromosome *mec* (SCC*mec*)—mediated resistance^19^. MRSA is a leading cause of antimicrobial resistance (AMR)-related mortality, with over 100,000 deaths in 2019^20^. While the incidence of AMR-linked deaths in children under five has decreased due to improved infection prevention strategies, the overall increase in MRSA-related mortality among older adults remains alarming^21^.

MRSA in fish and fishery products was unrecorded before 2009^22^. Since then, numerous studies on MRSA incidence in fish and fisheries products have emerged globally, raising concerns about seafood contamination and zoonotic potential^23,24^. To date, there has been little published information on the incidence and features of MRSA clones isolated from aquaculture environments, where fish processing and handling are limited. Hence, comprehensive surveillance of *S. aureus* genotypes in aquatic species and their contacts is essential for understanding global epidemiology and risks posed by MRSA clones. The current study explored the clonal variety of MRSA and MSSA clones isolated from aquatic shrimp and fish (saltwater or freshwater fish) as well as their close contacts. We also performed molecular typing utilizing a DNA microarray-based method to identify AMR genotypes and virulence factors, particularly enterotoxins, in the recovered isolates.

## Methods

### Farms and sample collection

**Fig. 1:**
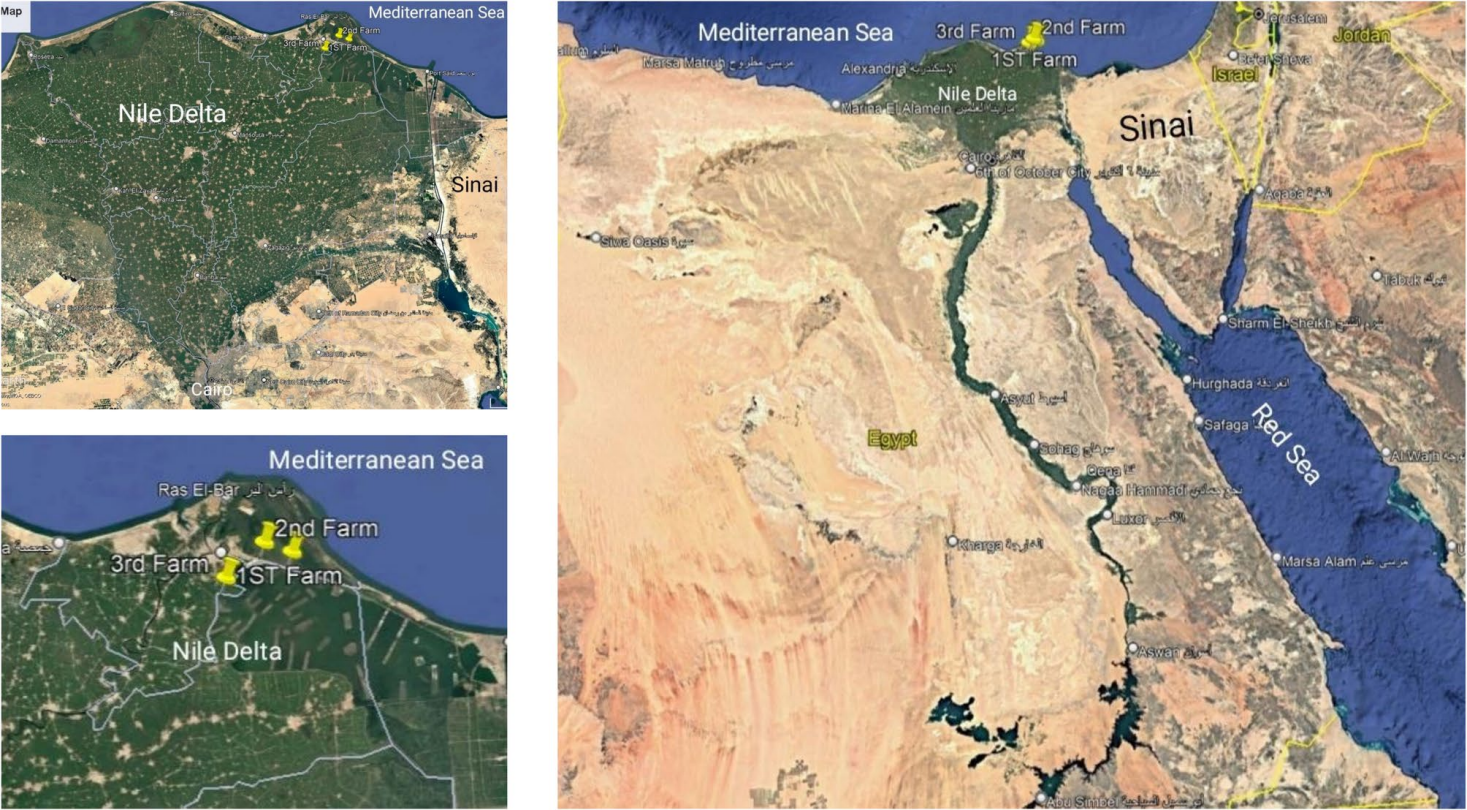
Map showing the collection sites in commercial aquaculture farms in Egypt. Map data: Google, ©2025 CNES/Airbus, Maxar Technologies. Map generated using Google Earth (https://earth.google.com/web/@29.15627819,30.35326787) (https://earth.google.com/web/@29.15627819,30.35326787,126.016527a,2726903.37421954d,35y,0h,0t,0r/data=CgRCAggBOgMKATBCAggASg0I____________ARAA?hl=ar).

The study involved three commercial aquaculture farms in Damietta Governorate on the Mediterranean coast of Egypt. The farms were selected based on convenience of samples collection. Farm (1), situated at 31.389742 longitude, 31.940483 latitude, cultivated shrimp (*Penaeus indicus*). Farm (2), located at 31.407287 longitude, 31.890132 latitude, raised marine fish, primarily sea bass (*Dicentrarcus labrax*) and sea bream (*Sparus aurata*). Farm (3), positioned at 31.355284 longitude, 31.821208 latitude, reared freshwater fish including African sharp tooth catfish (*Clarias garipienus*) and tilapia (*Oreochromis niloticus*) (Fig. 1). Detailed information regarding antibiotic and probiotic usage, water sources, pond dimensions, rearing systems, fish density (fish/m^3^), water exchange rates, feed types, pre-culture disinfection practices, and production rates per feddan (0.42 hectare) is presented in Supplementary File S1.

The study was conducted between December 2022 and May 2023 and encompassed 509 distinct samples, including shrimp (*n* = 150), 75 each of sea bass, sea bream, catfish, and tilapia, 15 water samples (five samples from each farm), and 22 samples each of hand and nasal swabs collected from farmworkers. The study follows the ARRIVE criteria for reporting in vivo investigations, which ensures transparent and complete reporting. We further affirm that all procedures follow the applicable norms and regulations outlined in the norms for the Care and Use of Agricultural Animals in Research and Teaching (3rd edition; http://www.fass.org/). The Agricultural Research Centre-Institutional Animal Care and Use Committee (ARC-IACUC) gave ethical authorization for the study (protocol ID: ARC, AHRI, 82, 24). Furthermore, consent from farm owners and agricultural workers to engage in the study and to carry out the research plan was secured.

### Sample collection and processing

#### Fish and shrimp

Fish and shrimp were caught by farm personnel using cast nets. Specimens that were free from visible skin lesions and of market size (≥ 200 g for tilapia, ≥ 250 g for catfish, ≥ 250 g for sea bream, ≥ 500 g seabass, and 30 g for shrimp), were selected and kept separately. They were transported alive in sterile plastic bags with air and water supply and transferred to the laboratory of Fish Diseases, Research and Management Unit, Damietta branch, Animal Health Research Institute, typically within a timeframe of about fifteen minutes, for bacteriological examinations.

Following euthanasia with MS-222 (Sigma, USA), the length and weight of each specimen were recorded. In the laboratory, fish and shrimp samples were aseptically processed in a biological safety cabinet. A macroscopic examination for any lesion or symptoms was also conducted. Swab samples were collected from fish mouths, vents, gills, and skin, as well as shrimp cuticles, in accordance with the International Commission on Microbiological Specifications for Food guidelines^27^. The swabs were immediately immersed in TSB containing 70 mg/ml NaCl and incubated at 37°C with agitation for 24 h. After incubation, a loopful from each inoculated broth was streaked onto Baird Parker agar (Oxoid, Hampshire, UK) and incubated at 37°C for 24–48 h^28^.

Twenty-five-gram samples of fish visceral organs (liver, kidney, and intestines), shrimp hepatopancreas, and fish and shrimp muscles were also obtained after skin sterilization with a hot spatula. Each sample was incorporated in a stomacher bag with 225 ml of peptone water (PW, pH 7.2) and homogenized at 14,000 rpm for 2.5 min. After incubating at 37°C overnight, 10 μL of each homogenate was streaked over a Baird Parker agar (Oxoid, Hampshire, UK) supplemented with 5% egg yolk potassium tellurite (Oxoid, Hampshire, UK) and incubated at 37°C for 24–48 h.

#### Farm water samples

Water samples were taken from fifteen sites, five at each farm, covering all fish and shrimp sampling locations as well as places away from the pond’s shoreline. Water was collected at a 5- to 10-cm depth at each site using a sterile 500 ml glass container, using the previously published method^29^. To ensure that all influent and effluent locations were represented, samples from each farm were pooled and carefully mixed. The samples were then transported to the laboratory in an insulated cooler. Upon arrival, individual samples were filtered through white gridded sterile mixed ester cellulose membranes (0.45 µm pore size/47 mm diameter), Merck, Burlington, Massachusetts, USA. The membranes were then placed on Baird-Parker enrichment agar plates supplemented with 5% egg yolk potassium tellurite (Oxoid, Hampshire, UK) and incubated at 37°C for 24–48 h. In general, the specific sampling steps and environmental conditions at the collection sites are visually documented in Supplementary Fig. S2.

#### Farm workers

Twenty-two each of hand and nasal swabs, respectively were collected from farm personnel (seven workers from farms 1 and 2, each, and eight persons from farm 3). Swab heads were pre-soaked in 4 ml of phosphate-buffered rinse solution (PBS) containing sodium citrate. Hand swabs were taken from the palm, interfinger gaps, fingertips, and nails, while nasal swabs were obtained by putting the swab into the nostrils and rotating it 360 degrees. All swab heads were carefully removed from the diluent, immediately packaged, and transferred to the laboratory in an insulated container for bacteriological investigation, using the same processing methodology as fish/shrimp skin swabs.

#### *Staphylococcus aureus* isolation

Typical staphylococcal colonies, characterized by their black, shiny, and convex appearance, were selected, purified on Baird-Parker agar, and incubated at 37°C for 24–48 h. The colonies were subsequently assessed for purity and subjected to biochemical assays, including the tube coagulase test (Becton, Dickinson, USA), catalase test, and mannitol fermentation test. Selected and re-cloned strains were preserved as glycerol stocks at -20°C for further analysis. Representative images of the colony characteristics and the outcomes of biochemical assays are provided in Supplementary Fig. S3 and S4.

### Matrix-assisted laser desorption ionization–time of flight mass spectrometry (MALDI-TOF MS)

All Baird-Parker positive cultures, presumptively identified as *S. aureus*, were verified using a MALDI-TOF MS Biotyper with a Microflex LT MALDI-TOF system, utilizing the Reference Library and Security Library databases (Bruker Daltonics, Bremen, Germany) in accordance with the manufacturer’s guidelines and the method described previously^30^.

### Lateral flow assays (LFA) for testing mecA/PbP2a

An experimental lateral flow culture confirmation test from Senova GmbH, Weimar, Germany, was used according to the manufacturer’s instructions. Approximately 1 µL of *S. aureus* culture was harvested from Columbia blood agar using an inoculation loop. This sample was mixed into the provided buffer and vortexed briefly. A second buffer was added, and 100 µL of the sample was pipetted into the test device’s sample well. The gadget was incubated at room temperature for 10 min, and the results were assessed visually. The presence of both, test and control lines, indicated a positive result. The existence of the control line alone predicted a negative result. A test without a control line was considered invalid. Detailed findings are present in Supplementary File S5.

### Microarray analysis: amplification, labelling, and array hybridization

*Staphylococcus aureus* was grown on Columbia blood agar for an overnight period at 37°C. A loop of staphylococcal cells (about 1–5 × 10^6^) was digested in a lysis reagent comprising lysozyme, lysostaphin, and RNAse A from the Genotyping-Kit—*S. aureus* (INTER-ARRAY by FZMB GmbH, Bad Langensalza, Germany, https://www.inter-array.com/INTER-ARRAY-Genotyping-Kit-S-aureus/). The DNeasy Blood & Tissue Kit (Qiagen GmbH, Hilden, Germany) was then used to lyse the samples and purify the DNA, as directed by the manufacturer. Linear amplification was carried out using one primer per target sequence. During this technique, biotin-16-dUTP was added to the amplicons, which were then tightly hybridized to specific probes on the microarray. The probes and primers used have previously been described^31^ and presented here as Supplementary File S6. All strains were genotyped using a microarray-based test (Genotyping Kit *S. aureus*; FZMB GmbH). A group of typical isolates was subsequently characterized using a secondary microarray to subtype their SCC*mec* elements, as published before^32^.

For the DNA microarrays, 336 unique targets were covalently affixed to the epoxy-coated plastic surface of eight-well microtiter strips. Biotin-labelled samples were hybridized in accordance with the manufacturer’s guidelines. After two washes, the samples were incubated for 60 min at 50°C and 550 rpm with the BioShake iQ device (Quantifoil Instruments GmbH (a BICO subsidiary) Jena, Germany). The microarrays were then washed for 10 min at 40 degrees Celsius and 550 rpm. Streptavidin horseradish peroxidase was used to detect hybridization by inducing local precipitation at amplicon binding sites. The microarrays were photographed and evaluated with a designated reader (FZMB GmbH). Clonal complex (CC) affiliations, SCC*mec* types, and epidemic strain identifications were identified by comparing hybridization profiles to the reference database^31^.

### Statistical analysis

The data were analyzed with the Statistical Package for Social Science (SPSS) application for Windows (Standard version 26). Quantitative data were summarized with frequencies and percentages.

## Results

In this study, 46 out of 509 specimens tested positive for *S. aureus*, representing 9.03% of the total. Of these, 34 specimens yielded a single isolate each, 10 specimens yielded two genotypically distinct isolates each, and two specimens yielded three distinct isolates each. In total, 60 *S. aureus* isolates (including MRSA) were recovered from the positive specimens. The isolates comprised 46 MRSA and 14 MSSA. In all cases, there was concordance of the results of genotypic results for *mecA* by microarray and phenotypic test results for PbP2a according to LFA.

Marine fish yielded the highest number of *S. aureus* isolates (17/150; 11.3%), followed by freshwater fish (12/150; 8.0%) and shrimp (10/150; 6.7%). Only 14 specimens of marine fish (9.3%), 8 specimens of freshwater fish (5.3%), 7 specimens of shrimp (4.6%), four samples of pooled water (26.7%), and 13 samples of close contact workers (29.5%) tested positive for *S. aureus.* These positive specimens yielded 17 *S. aureus* isolates from marine fish, 12 from freshwater fish, 10 from shrimp, 4 from pooled water samples, and 17 from contact workers. The MRSA carriage rate was 8.6% (13/150) in marine fish, 6.6% (10/150) in freshwater fish, and 4.7% (7/150) in shrimp. For farm workers, the MRSA carriage rate ranged between 0.0% (0/16) in freshwater farm workers and 42.9% (6/14) in shrimp and marine water farm workers. The ratio of MRSA among the recovered *S. aureus* isolates was 7/10 in shrimp, 13/17 in marine fish, and 10/12 in freshwater fish, while it was 0.0 among the workers of freshwater farm, 6/10 of shrimp worker’s and 6/7 of marine farm workers. On the other side, the rates of MSSA carriage among the tested specimens were 2% (3/150) in shrimp, 2.6% (4/150) in marine fish, 1.3% (2/150) in freshwater fish, 28.5% (4/14) in shrimp farm workers, and 7.1% (1/14) in saltwater farm workers. No MSSA isolates were found in other samples (Table 1).

**Table 1:** Distribution of samples harbouring *Staphylococcus aureus* isolates (MSSA and MRSA) in the investigated aquaculture farms.

Clonal lineages	Farm 1			Farm 2			Farm 3		
	Farm water (*n* = 5)	Shrimp (*n* = 150)	Workers (*n* = 14)	Farm water (*n* = 5)	Marine fish (*n* = 150)	Workers (*n* = 14)	Farm water (*n* = 5)	Freshwater fish (*n* = 150)	Workers (*n* = 16)
CC88-MRSA (*n* = 32)	3	6	4	–	8	5	1	5	–
CC361-MSSA (*n* = 3)	–	1	–	–	–	1	–	1	–
CC361-MRSA (*n* = 10)	–	–	1	–	5	1	–	3	–
CC1-MSSA (*n* = 11)	–	2	4	–	4	–	–	1	–
CC15-MRSA (*n* = 3)	–	1	–	–	–	–	–	2	–
CC152-MRSA (*n* = 1)	–	–	1	–	–	–	–	–	–
Samples positive for *S. aureus* (*n*) %	(3) 60%	7 (4.7%)	6 (42.9%)	0 (0)	14 (9.3%)	7 (50%)	1 (20%)	8 (5.3%)	0 (0)
Total of *S. aureus* isolates (*n*) %	(3) 60%	10 (6.7%)	10 (71.4%)	0 (0)	17 (11.3%)	7 (50%)	1 (20%)	12 (8.0%)	0 (0)
MRSA recovered (*n*) %	(3) 60%	7 (4.7%)	6 (42.9%)	0 (0)	13 (8.6%)	6 (42.9%)	1 (20%)	10 (6.6%)	0 (0)
MSSA recovered (*n*) %	0(0)	3 (2%)	4 (28.5%)	0 (0)	4 (2.6%)	1 (7.1%)	0 (0)	2 (1.3%)	0 (0)

Hand swabs showed the highest *S. aureus* carriage rate (10/22; 45.5%), followed by nasal swabs (7/22; 31.8%), farm water samples (4/15; 25.7%), skin swabs (14/150; 9.30%), muscle specimens (13/150; 8.7%), and visceral organ specimens (12/150; 8%) (Table 2). The most common sources of MRSA were hand swabs (7/22; 31.8%), followed by farm water samples (4/15; 26.7%), nasal swabs 5/22; 22.7%), skin swabs (11/150; 7.3%); muscle specimens 10/150; 6.6%) and visceral organ specimens (9/150; 6.0%).

**Table 2:** Strain affiliations and the origins of *Staphylococcus aureus* isolates (MSSA and MRSA).

Strains affiliation	Origin of samples					
	Fish/shrimp farms				Co-workers	
	Water samples (*n* = 15)	Skin swabs (*n* = 150)	Muscles (*n* = 150)	Viscera (*n* = 150)	Hand swabs (*n* = 22)	Nasal swabs (*n* = 22)
CC88-MRSA-[IV + fus] (*n* = 32)	4 F1 (*n* = 3) F3 (*n* = 1)	5 F2 (*n* = 3) F3 (*n* = 2)	8 F1 (*n* = 3), F2 (*n* = 3), F3 (*n* = 2)	6 F1 (*n* = 3), F2 (*n* = 2), F3 (*n* = 1)	5 F1 (*n* = 3) F2 (*n* = 2)	4 F1 (*n* = 1) F2 (*n* = 3)
CC361-MSSA (*n* = 3)	–	1 F3	–	1 F1	–	1 F2
CC361-MRSA-V/VT, "WA MRSA-70/110" (*n* = 10)	–	4 F2 (*n* = 2) F3 (*n* = 2)	1 F2	3 F2 (*n* = 2) F3 (*n* = 1)	1 F2	1 F1
CC1-MSSA (*n* = 11)	–	2 F2 (*n* = 1) F3 (*n* = 1)	3 F1 (*n* = 1) F2 (*n* = 2)	2 F1 (*n* = 1) F2 (*n* = 1)	3 F1	1 F1
CC15-MRSA-[V + fus] (*n* = 3)	–	2 F3	1 F1	–	–	–
CC152-MRSA-[V + fus] (*n* = 1)	–	–	–	–	1 F1	–
MRSA	4 (26.7)	11 (7.3%)	10 (6.6%)	9 (6.0%)	7 (31.8%)	5 (22.7%)
MSSA	0 (0%)	3 (2.0%)	3 (2.0%)	3 (2.0%)	3 (13.6%)	2 (9.0%)
Total (*n* = 60)	(*n* = 4) 26.6%	(*n* = 14) 9.30%	(*n* = 13) 8.7%	(*n* = 12) 8.0%	(*n* = 10) 45.5%	(*n* = 7) 31.8%

F1: Shrimp farm.

F2: Marine fish farm.

F3: Freshwater fish farm.

Four different CCs of MRSA and two CCs of MSSA were identified. The most abundant MRSA strains were CC88-MRSA-[IV + *fus*] (*n* = 32), followed by CC361-MRSA-V/VT, "WA MRSA-70/110" (*n* = 10), CC15-MRSA-[V + *fus*] (*n* = 3) and CC152-MRSA-[V + *fus*] (PVL +) (*n* = 1); while the two identified CCs of MSSA were CC1 (*n* = 11) and CC361 (*n* = 3) (Tables 1 and 2). The great majority of MRSA and MSSA strains were observed among both human and aquaculture specimens (Tables 1 and 2). The detailed genotypic information of all clones identified as well as their resistance profile and the virulence determinants are illustrated in Tables 3, 4, and 5; and Supplementary File S7 and summarized as follows:

**Table 3:** Genomic markers and the abundance of antibiotic resistance genes of the recovered *S. aureus* clones (*n* = 60).

Clonal lineages	CC88 MRSA (*n* = 32)	CC361-MSSA (*n* = 3)	CC361-MRSA (*n* = 10)	CC1-MSSA (*n* = 11)	CC15-MRSA (*n* = 3)	CC152-MRSA (*n* = 1)
Capsular type	8	8	8	8	8	5
*agr*	III	I	I	III	II	I
SCC*mec* typing	*ccr A/B-2*	–	*ccrAA* and *ccrC*	–	*ccrAA* and *ccrC*	*ccrAA* and *ccrC*
*mecA*	+	–	+	–	+	+
*bla*Z*/bla*I*/bla*R	+	+(2), − (1)	+	+(1), − (10)	+	-
*tet*(K)	+	–	–	–	–	–
*tet*(M)	+(1)	–	–	–	–	–
*msr*(A)	–	–	+	–	–	–
*mpB*(C)	–	–	+	–	–	–
*fusC*	+	–	–	–	+	+
*aphA3*	–	–	+	–	–	–
*sat*	–	–	+	–	–	–
*aacA-aphD*	–	–	+	–	+	+
*fos*B	–	+	+	–	+	–
*aad*D	–	–	–	–	+	–
*sdr*M	+	+	+	+	+	+

*SCCmec:* Staphylococcal cassette chromosome *mec*; *ccr A/B-2:* cassette chromosome recombinase genes *A/B-2; ccrAA and ccrC*: cassette chromosome recombinase genes “*ccrAA*” (hypothetical) and *ccrC; bla*Z*: beta-lactamase; bla*I* :* beta lactamase repressor (inhibitor)*; bla*R*:* beta-lactamase regulatory protein; *tet*(K) and *tet*(M)*:* tetracycline-resistance; *msr*(A)*:* energy-dependent efflux of erythromycin; *mpbBM* (= *mpB*(*C*)): probable lysylphosphatidyl-glycerol synthetase*; fus*C*:* hypothetical protein associated with fusidic acid resistance*; aphA3:* neomycin/kanamycin resistance*; sat:* streptothricine-acetyl-transferase*; aacA-aphD:* bifunctional enzyme Aac/Aph, gentamicin resistance; *fosB:* metallothiol transferase; *aadD:* tobramycin resistance; *sdrM:* transport-/efflux-protein.

**Table 4:** Prevalence of enterotoxins, leukocidins and hemolysins of the recovered *S. aureus* clones (*n* = 60).

Clonal complex	CC88- MRSA	CC361-MSSA	CC361-MRSA	CC1-MSSA	CC15-MRSA	CC152-MRSA
	(*n* = 32)	(*n* = 3)	(*n* = 10)	(*n* = 11)	(*n* = 3)	(*n* = 1)
*sea*	–	–	–	–	–	–
*seb*	–	–	–	–	–	–
*sec*	–	–	–	–	–	–
*sed*	–	–	–	–	–	–
*see*	–	–	–	–	–	–
*sej*	–	–	–	–	–	–
*sel*	–	–	–	–	–	–
*ser*	–	–	–	–	–	–
*seq*	–	–	–	–	–	–
*sek*	–	–	–	–	–	–
*seh*	–	–	–	+	–	–
*sei*	–	+	+	–	–	–
*sem*	–	+	+	–	–	–
*sen*	–	+	+	–	–	–
*seo*	–	+	+	–	–	–
*egc* cluster	–	+	+	–	–	–
*seu*	–	+	+	–	–	–
*seg*	–	+	+	–	–	–
*sep*	+	–	–	–	–	–
*lukF/lukS*	+	+	+	+	+	+
*lukX/lukY*	+	+	+	+	+	+
*lukD/lukE*	+	+	+	+	+	–
*lukS-PV*	–	–	–	–	–	–
*lukF-PV*	–	–	–	–	–	–
*hl*	+	+	+	+	+	–
*hlgA*	+	+	+	+	+	+
*hla*	+	+	+	+	+	+
*hlb*	+	+	+	+	–	–
*sak*	+	+	+	– (+ 1)	–	+
*chp*	+	+	–	–	+	–
*scn*	+	+	+	– (+ 1)	+	+
*splA*	+	+	–	+	+	–
*splB*	+	+	–	+	+	–
*splE*	–	–	–	+	+	–

*se*: Staphylococcal enterotoxins (*a, b, c, d, e, j, l, r, q, k, h, i, e, m, n,o, u, g, p*); *luk*: leukocidin; *hl:* putative membrane protein*; hla:* hemolysin alpha*; hlb:* hemolysin beta*; hlgA:* hemolysin gamma, component; *sak*: staphylokinase; *chp*: chemotaxis-inhibiting protein; *scn*: staphylococcal complement inhibitor; *splA*: serin- protease A; *splB*: serin- protease B*;splE*: serin- protease E.

**Table 5:** Biofilm and adhesion associated genes of the recovered *S. aureus* clones (*n* = 60).

Gene	CC88 MRSA	CC361–MSSA	CC361–MRSA	CC1–MSSA	CC15–MRSA	CC152–MRSA
	(*n* = 32)	(*n* = 3)	(*n* = 10)	(*n* = 11)	(*n* = 3)	(*n* = 1)
*icaA*	+	+	+	+	+	+
*icaC*	+	+	+	+	+	–
*icaD*	+	+	+	+	+	+
*bap*	–	–	–	–	–	–
*clfA*	+	+	+	+	+	+
*clfB*	+	+	+	+	+	+
*cna*	–	–	–	+	–	+
*ebpS*	+	+	+	+	+	+
*eno*	+	+	+	+	+	+
*fnbA*	+	+	+	+	+	+
*fnbB*	+	+	+	+	+	+
*map*	+	+	+	–	+	–
*sasG*	+	+	+	+	+	–
*sdrC*	+	+	+	+	+	–
*sdrD*	+	+	+	+	+	+
*vwb*	+	+	+	+	+	+
*bbp*	+	+	+	+	+	+

*icaA*: intercellular adhesion protein A; *icaC*: intercellular adhesion protein C; *icaD*: biofilm PIA synthesis protein D; *bap*: surface protein involved in biofilm formation; *clfA*: clumping factor A; *clfB*: clumping factor B; *cna*: collagen-binding adhesin; *ebpS*: cell surface elastin binding protein; *fnbA*: fibronectin-binding protein A; *fnbB*: fibronectin-binding protein B; *map*: Major histocompatibility complex class II analog protein (= Extracellular adherence protein, *eap*); *sasG*: *Staphylococcus aureus* surface protein G; *sdrC*: Ser-Asp rich fibrinogen-/bone sialoprotein-binding protein C; *sdrD*: Ser-Asp rich fibrinogen-/bone sialoprotein-binding protein D; *vwb*: van Willebrand factor binding protein; *bbp*: bone sialoprotein-binding protein.

### Clonal complex 88

A total of 32 isolates belonging to CC88 were identified, all CC88-MRSA-[IV + *fus*]. This clonal group was detected across all sample types, including human swabs. Isolates of this clonal complex originated primarily from shrimp farm (*n* = 13), marine fish farm (*n* = 13), and freshwater fish farm (*n* = 6). The main source of this clonal lineage was marine fish (*n* = 8) followed by shrimp (*n* = 6), freshwater fish (*n* = 5), farms workers (*n* = 9) and farm water samples (*n* = 4). Muscle specimens were the most abundant sources of this clonal lineage (*n* = 8) followed by visceral organs (*n* = 6), skin swabs (*n* = 5), hand swabs (*n* = 5) while the least providers were water samples (*n* = 4) and nasal swabs (*n* = 4) (Tables 1 and 2).

The isolates exhibited genomic markers characteristic for CC88, including accessory gene regulator (*agr*) III, and capsule type 8. The cassette chromosome recombinase genes (*ccr*) A/B-2 genes were present in addition to *mec*A and *fus*C. The application of the second array on three isolates indicated a presence of *cstB-*SCC2 (Q2G1R6; GenBank BA000033.2:[55,346...56,663]) indicating that the composite SCC*mec/fus* element was derived from SCC*mec* IVa. Additionally, these isolates harbored genes encoding the beta-lactamase operon *bla*Z*/bla*I*/bla*R as well as tetracycline-resistance genes *tet*(K) and *tet*(M). Except for *sep*, no other enterotoxin genes were found. The isolates contained leukocidin genes (*luk*F*/*S*, luk*X*/*Y*, luk*D*/*E), hemolysin genes (*hl, hla, hlb, hlg*A), and phage-associated virulence genes *sak, chp, scn*. They also possessed genes responsible for biofilm production such as the *ica*-locus but excluding the surface protein involved in biofilm formation (*bap*) and collagen-binding adhesin (*cna*), along with adhesion-related genes.

### Clonal complex 361

Thirteen isolates of CC361 were identified with three isolates being MSSA, while ten isolates were MRSA (CC361-MRSA-V/VT, resembling "WA MRSA-70/110"). The three isolates of MSSA originated from a skin swab of freshwater fish (*n* = 1), from the viscera of a shrimp (*n* = 1) and a nasal swab of worker in a marine fish farm (*n* = 1); while MRSA clones came from human and animal samples but not from any water samples collected. The most common source of isolates of this clone was marine fish (*n* = 5), followed by freshwater fish (*n* = 3) and human nasal and hand swabs (*n* = 1 each) from shrimp and marine farms, respectively (Tables 1 and 2).

Both MRSA and MSSA clones were characterized by the presence of *agr* I, and capsule type 8. The MSSA isolates were *mecA*-negative, while two of them carried beta-lactamase operon genes *bla*Z*/bla*I*/bla*R. MRSA isolates were assigned to SCC*mec* V or VT harboring *mec*A, *ccr*AA and *ccr*C. Two representative isolates were subtyped using the second array and shown to have SCC*mec* VT elements based on the presence of the gene encoding D1GU38 (GenBank AM990992.1 [35,035…35,898]) that accompanies the second set of *ccrAA/ccrC* recombinase genes in composite and SCC*mec* VT elements.

MRSA isolates carried multiple AMR genes, such as *mec*A, *bla*Z*/bla*I*/bla*R*,* macrolide resistance genes [*msr*A and *mpB(C)*], a neomycin/kanamycin resistance gene (*aph*A3)*,* the streptothricin-acetyl transferase gene (*sat*)*,* a gentamicin resistance gene (*aac*A*-aphD*)*,* as well as *sdr*M gene encoding an efflux pump. Isolates of this clonal complex harbored the *egc* cluster enterotoxins (*sei, sem, sen, seo, seu* and *seg)*; leukocidins genes (*luk*F/*S, luk*X*/*Y*,* *luk*D*/*E) and hemolysin genes (*hl, hla, hlb, hlg*A*).* The *sak* and *scn* genes were always present, while the *chp* gene was present only in MSSA ones. Virulence determinants serin protease A (*spl*A) and serin protease B (*spl*B*)* were also present in MSSA isolates. Isolates of this clonal lineage also harbored all genes responsible for biofilm production and adhesion to host molecules (except *bap* and *cna)*.

### Clonal complex 1

Eleven isolates of this clonal lineage were recovered. The isolates were all MSSA and originated primarily from marine fish (*n* = 4), farm workers of shrimp (*n* = 4; 3 from hand swabs and one from a nasal swab), shrimp samples (*n* = 2; i.e., one from muscle and one from a visceral organ specimen) and a skin swab of freshwater fish (*n* = 1) (Tables 1 and 2). The isolates were characterized by the presence of *agr* III, and capsule type 8. While all isolates were *mec*A negative and carried the *sdr*M gene, one isolate harbored gene encoding *bla*Z*/bla*I*/bla*R. All isolates harbored the enterotoxin H gene *seh*, leukocidin genes (*luk*F/S*, luk*X*/*Y*,* *luk*D/E*) *and hemolysin genes (*hl, hla, hlb, hlg*A*)* but lacked *sak* and *scn* genes except one. Virulence determinants including *spl*A*, spl*B *and spl*E were present (Table 4). The isolates harbored all genes responsible for biofilm production (except *bap)* and adhesion associated genes but lacked major histocompatibility complex class II analog protein *(map)* gene (Table 5).

### Clonal complex 15

Three isolates of this clonal lineage were identified from two skin swabs of freshwater fish and from one shrimp muscle specimen (Tables 1 and 2). No isolates from this clonal lineage came from human samples. Isolates of this clone were characterized by the presence of *agr* II, and capsule type 8; and harbored *ccr*AA and *ccr*C. The isolates were affiliated to 15-MRSA-[V + *fus*] and harbored *mec*A*, bla*Z*/ bla*I*/bla*R*, aad*D*, aac*A*-aph*D*, fus*C and *sdr*M genes (Table 3). The application of the second array ruled out the presence of a SCC*mec* VT-derived element based on absence of D1GU38.

The isolates of this clone carried no enterotoxins but carried leukocidins (*luk*F/S*, luk*X/Y*,* *luk*D/E*),* hemolysins* (Hl, hlg*A*, hla),* and phage associated genes* (chp* and *scn)* while the *sak* gene was absent from all isolates. Virulence determinants including *spl*A*, spl*B *and spl*E were all present in all isolates (Table 4). Isolates of this clonal lineage also harbored all genes responsible for biofilm production (except *bap*, which was not found in a single case) and adhesion associated genes* (cna* gene) (Table 5).

### Clonal complex 152

One isolate of this clonal lineage was recovered from a hand swab of shrimp farm worker (Table 1&2). Neither shrimp nor fish carried this clonal complex. This isolate was characterized by the presence of *agr* I, and capsule type 5. It was assigned to CC152-MRSA-[V + *fus*] and harbored *mec*A, *ccr*AA, *ccr*C, *aac*A*-aph*D*, fus*C*,* and *sdr*M genes (Table 3). The second array ruled out the presence of a SCC*mec* VT-derived element based on absence of D1GU38. The isolate carried no enterotoxins but carried leukocidins (*luk*F/S*, and luk*X/Y*),* hemolysins* (hlg*A*,* and *hla),* and phage associated genes* (sak* and *scn*), while the gene *chp* was absent from all isolates. Virulence determinants including *spl*A*, spl*B *and spl*E were all absent (Table 4). It also lacked PVL genes. Isolate of this clonal lineage also harbored all genes responsible for biofilm production (except for *ica*C*, & bap* genes), and adhesion associated genes [except for *S. aureus* surface protein G (*sas*G) and Ser-Asp rich fibrinogen-/bone sialoprotein-binding protein *C (sdr*C)] (Table 5).

## Discussion

Aquaculture is recognized as a reservoir for resistant infections, but MRSA origins remain unclear^1^. To our knowledge, the characteristics of MRSA clones recovered from fishery environments are poorly understood^26,33^. This study provides a thorough investigation of a diverse set of *S. aureus/*MRSA clones collected from fish and shrimp aquaculture farms in the Nile Delta region of Egypt. We used MALDI-TOF MS, an experimental PbP2a-LFA and DNA-microarrays to analyse 60 *S. aureus* isolates from shrimp, marine and freshwater fish, as well as individuals in close contact with them and farm water.

In Egypt, there has been limited information regarding MRSA colonization in aquatic species, with most investigations focusing on retail market samples. In this study, all MRSA isolates carried multiple AMR genes and virulence factors. Furthermore, MSSA and MRSA were detected at relatively high rates in fish & shrimp, and at very high rates in the farm workers. Similar prevalence patterns have been reported in Egypt^24^, the Middle East^25^, Asia^9^. However, even higher MRSA frequencies were observed elsewhere^26^. The presence of MRSA isolates in the examined healthy fish and shrimp could be as a source of MRSA dissemination in humans, or it could be a consequence of MRSA colonisation of farm personnel. Recently, a meta-analysis of 57 studies showed wide variability in MRSA prevalence (0–100%, mean 11.7%) influenced by sampling, geography, and species^34^. While MRSA detection in water is rare^35^, persistence has been documented in seawater and rivers^36,37^. In our study, 26.6% of water samples were MRSA-positive, suggesting a role of the higher density of host organisms in aquaculture compared to natural settings as well as of multiple infection sources such as utensils, human activities, fishing, the origin of the fish, or other substrates, reservoirs, or carriers, which warrant further exploration. The transmission from humans to fish is a likely pathway rather than the adaptation of *S. aureus* to its host^29^.

The genotypic characteristics of MRSA clones colonizing shrimp, fish, and their environments in aquaculture farms remain largely unexplored. Our investigation, employing molecular characterization techniques, identified five distinct CCs. CC88 emerged as the predominant lineage, followed by CC1, CC361, CC15, and CC152. The CC88 lineage, specifically CC88-MRSA-[IV + *fus*], accounted for 50% of all isolates and isolated for the first time in Egypt from marine and freshwater fish, shrimp, and their respective farm waters. This clonal lineage was previously identified in Egypt from mastitic milk of household cattle^38^ and subsequently in healthy Egyptian dairy cattle, buffalo, and their caretakers^28^ and has also been observed in various food products, including marine fish^40,41^. Notably, the CC88 MRSA isolates reported in this study exhibited multidrug resistance but lacked enterotoxin genes, except for the *sep* gene which is considered as a superantigen being identified in cases of staphylococcal food poisoning even without the presence of classic staphylococcal enterotoxins^39^^.^ The occurrence of these lineages in aquatic organisms and their habitats raises concerns about the potential transmission of antibiotic-resistant bacteria to humans.

Here, we describe a rarely reported clone from CC361, which has never been observed in aquatic fisheries in Egypt. This clonal lineage constituted 20.3% of all the isolates. The most common sources of this clonal lineage were marine fish and their workers, followed by freshwater fish. The MRSA isolates were as assigned to CC361-MRSA- VT, a strain similar or identical to the one previously described in Australia as “WA MRSA-70/110,” and were multi-drug resistant (MDR) strains. Both, MSSA and MRSA clones harboured the *egc* cluster enterotoxins, leukocidin genes*,* and hemolysin genes, but were negative for the PVL genes. Some of the enterotoxins encoded by the *egc* cluster of *S. aureus* have proven emetic activity and might be related to food poisoning, indeed, even in absence of the classic enterotoxin genes^15^. This lineage was earlier found in Egyptian cattle^28^ and human patients in Gulf countries^42–44^.

CC1 constituted 18.3% of all isolates recovered and these isolates belonged to CC1-MSSA. Aquaculture shrimps and their workers were the primary sources of these isolates, followed by marine fish, while freshwater fish represented the least abundant source. CC1 is known to be associated with humans^31^ and livestock^13^, but an observation in fish and shrimp is rather new although its presence has already been noted in aquatic environments, particularly in landing centres and high-salinity areas^40^. The presence of this clonal lineage in shrimp and marine fish could potentially pose a risk of foodborne illness associated with the consumption of raw, contaminated fish and shrimp and represents a potential occupational hazard for workers in the food production chain. Furthermore, all CC1 isolates carried the enterotoxin H gene which has been observed to cause food poisonings^45–47^.

CC15 MSSA is globally common^31^ and it has also been found in Egypt, in a cow with mastitis^38^. In contrast, CC15-MRSA used to be extremely rare until it emerged during the last decade, in humans and in livestock in Saudi-Arabia, and across the Middle East^43^ and Egypt^28^. The presence of CC15-MRSA in shrimp and freshwater fish environments raises concerns regarding potential zoonotic transmission and foodborne transmission of MRSA but could also be a consequence of anthropo-zoonotic transmission.

A hand swab from a shrimp worker yielded an isolate of clonal complex 152, which demonstrated multidrug resistance and lacked enterotoxin genes. This isolate was identified as CC152-MRSA-[V + *fus*]. CC152 has been documented in the Gulf region^43^, and in general, CC152 is prevalent throughout Africa, especially in West African nations, and in countries with populations of African heritage, such as Trinidad. The initial detection of this lineage in Egypt was observed in clinically healthy dairy cattle, buffalo, and their handlers^28^ and an albeit PVL-positive, isolate with an apparently identical SCC*mec* element has been sequenced from a hospital patient from Egypt (GenBank: JAEOUX01)^48^. Overall, the presence of classical MDR-human CCs in aquaculture suggests human-to-environment transmission. Multiple pathways, including hospital effluents, livestock waste, and wastewater, contribute to resistant pathogen spread^34,49^. Surface water bodies, particularly rivers, can serve as key storage locations for effluent, contaminants, antibiotic-resistant microorganisms, and antibiotic resistance genes. These environmental circumstances may increase the proliferation and stability of antibiotic-resistant bacteria, allowing the interchange of resistance determinants^50^.

Although this study provides valuable insights into the prevalence and genetic characterization of MRSA in commercial aquaculture farms, certain limitations should be acknowledged. The sampling size, while sufficient to detect MRSA occurrence and perform molecular analyses, may not fully capture the overall diversity of *S. aureus* strains present across all aquaculture systems. In addition, the geographic coverage was restricted to selected commercial farms in one Governorate, and therefore the findings may not be fully representative of aquaculture operations in other regions of Egypt. From a methodological perspective, the study relied on culture-based isolation followed by molecular detection of resistance and virulence genes; while these approaches are widely accepted and robust, they may not detect non-culturable or low-abundance strains. On the other hand, they are less prone to contamination. Consequently, the results should be interpreted within the context of these constraints. Future studies incorporating larger sample sizes, broader geographic coverage, and complementary high-throughput or metagenomic approaches would further strengthen surveillance of MRSA in aquaculture environments.

## Conclusion

This study has demonstrated that shrimp, as well as marine and freshwater fish, can act as reservoirs for antimicrobial-resistant *S. aureus* (including MRSA), in aquaculture farms in Egypt. MRSA clones of the identified CCs were MDR and contained various genes encoding virulence factors, such as leukocidins, hemolysins, and proteases. Additionally, these clones possessed genes associated with adhesion and biofilm formation, although the prevalence of enterotoxins was low. Notably, isolates of CC361 harbored the *egc* cluster enterotoxins, while CC1 carried the enterotoxin H gene. Notably, not a single PVL-positive isolate was identified although PVL genes are very common among Egyptian *S. aureus* and MRSA. The presence of MRSA clones in aquatic ecosystems could represent a significant route for the transmission and spread of multiple pathogenic factors to human populations and emphasize the need to expand monitoring the AMR in the study regions. A better understanding of *S. aureus* spill-over dynamics in aquaculture systems will mitigate future risks to fish and human health. Our findings highlight the importance of monitoring MRSA within aquaculture systems in Egypt.

## Recommendations

Given the detection of MRSA in shrimp and fish farms, we recommend the following measures: establish routine screening programs for MRSA in aquaculture farms, and among their personnel, to detect and control outbreaks early. Strengthen farm-level hygiene, water quality management, and handling protocols to reduce bacterial transmission. Limit the use of antibiotics in aquaculture and promote alternative strategies such as probiotics and vaccination to minimize selective pressure for resistance. Encourage collaboration between veterinary, aquaculture, and public health authorities to monitor zoonotic transmission risks and conduct longitudinal studies to assess the persistence and genetic evolution of MRSA in aquaculture environments. These recommendations underscore the need for a One Health approach, integrating aquaculture management with broader antimicrobial resistance control strategies.

## Supplementary Information

Legend of Supplementary files.

Supplementary Information 1.

Supplementary Information 2.

Supplementary Information 3.

Supplementary Information 4.

Supplementary Information 5.

Supplementary Information 6.

Supplementary Information 7.

## Data Availability

All data generated or analyzed during this study are included in this published article [and its supplementary information files.
