# Supplementary material for: Prevalence and genetic characterization of methicillin-resistant Staphylococcus aureus in Commercial aquaculture farms in Egypt
Source: Sci Rep. 2026 Apr 10;16:12026. doi: 10.1038/s41598-026-40144-y (PMC13068896; doi:10.1038/s41598-026-40144-y)
Supplement: Supplementary file 1 — Legend of Supplementary files. [file 41598_2026_40144_MOESM1_ESM.docx]

**Legend of Supplementary files**

**Supplementary File S1:** Epidemiological data of the farms examined.

**Supplementary Figure S2:** Photographs detailing the sampling steps at the aquaculture farms.

**Supplementary Figure S3:** High-resolution images showing the typical colony characteristics of *S. aureus* on Baird-Parker agar.

**Supplementary Figure S4:** Visual results of the biochemical assays confirming the identification of the isolates.

**Supplementary File 5:** Detailed LFA findings and MALDI-ToF.

**Supplementary File 6:** The probes and primers used in this study.

**Supplementary File 7:** Detailed genotypic information of all clones identified as well as their resistance profile and the virulence determinants.
