# Supplementary material for: Prevalence and genetic characterization of methicillin-resistant Staphylococcus aureus in Commercial aquaculture farms in Egypt
Source: Sci Rep. 2026 Apr 10;16:12026. doi: 10.1038/s41598-026-40144-y (PMC13068896; doi:10.1038/s41598-026-40144-y)
Supplement: Supplementary file 3 — Supplementary Information 2. [file 41598_2026_40144_MOESM3_ESM.pdf]

Supplementary Figure S2.
Sampling procedures in commercial aquaculture farms. Photographs illustrating the sample collection workflow conducted in this study.

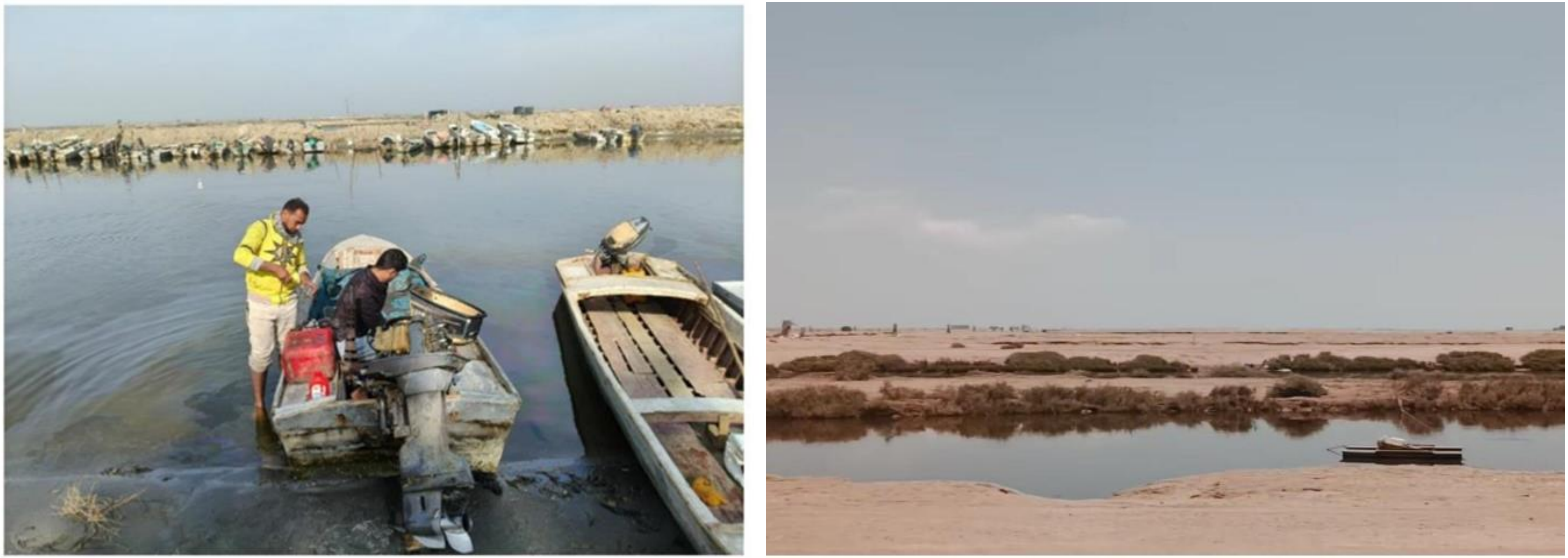

(a) General view of the commercial aquaculture ponds selected for sampling in Egypt.

Supplementary Figure S2 (continue)

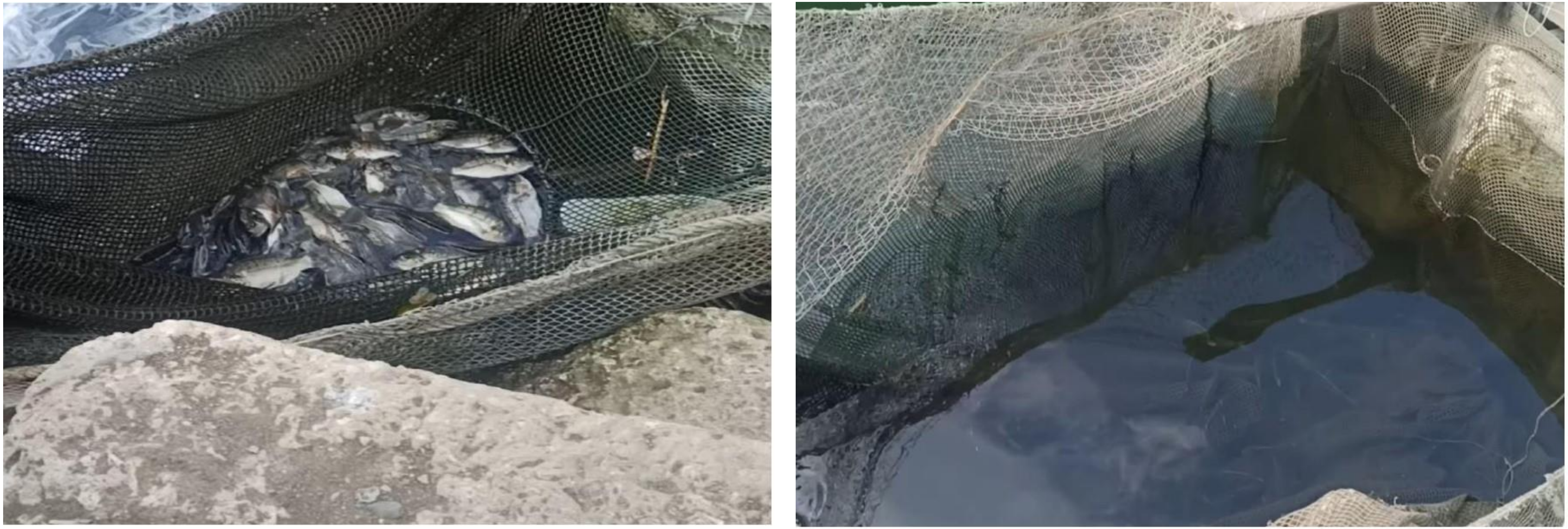

(b) Collection of samples using cast nets.

Supplementary Figure S2 (continue)

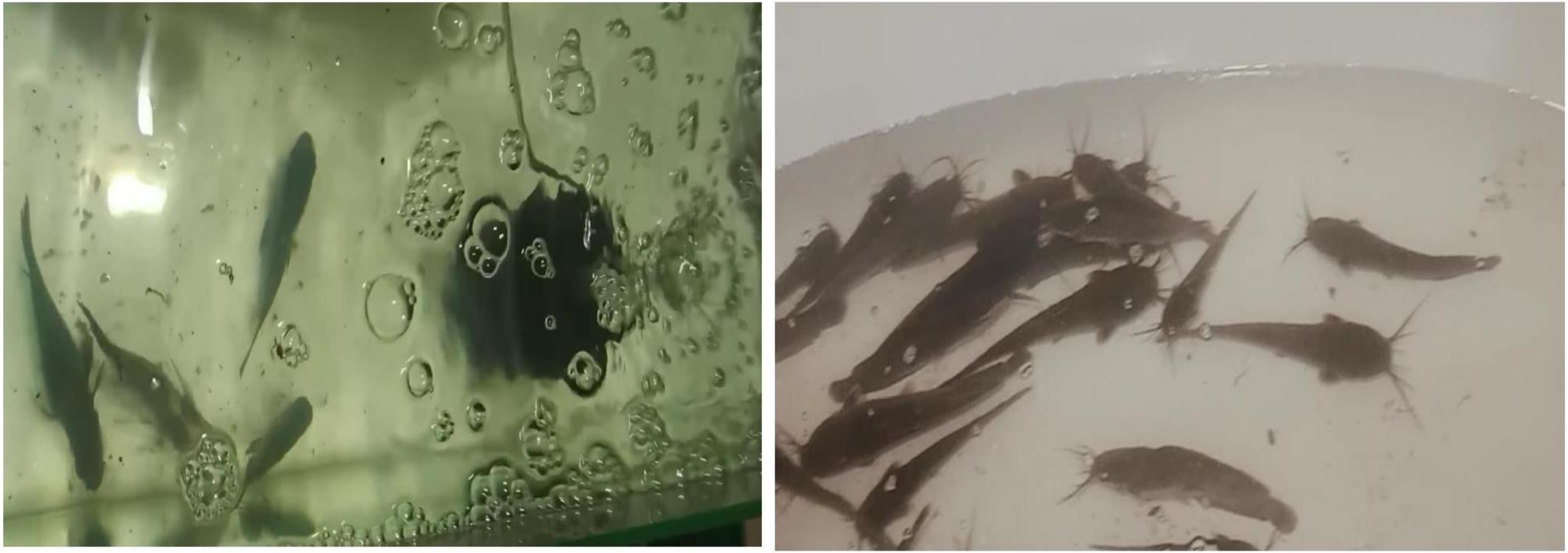

(c) Samples transportation

Supplementary Figure S2 (continue)

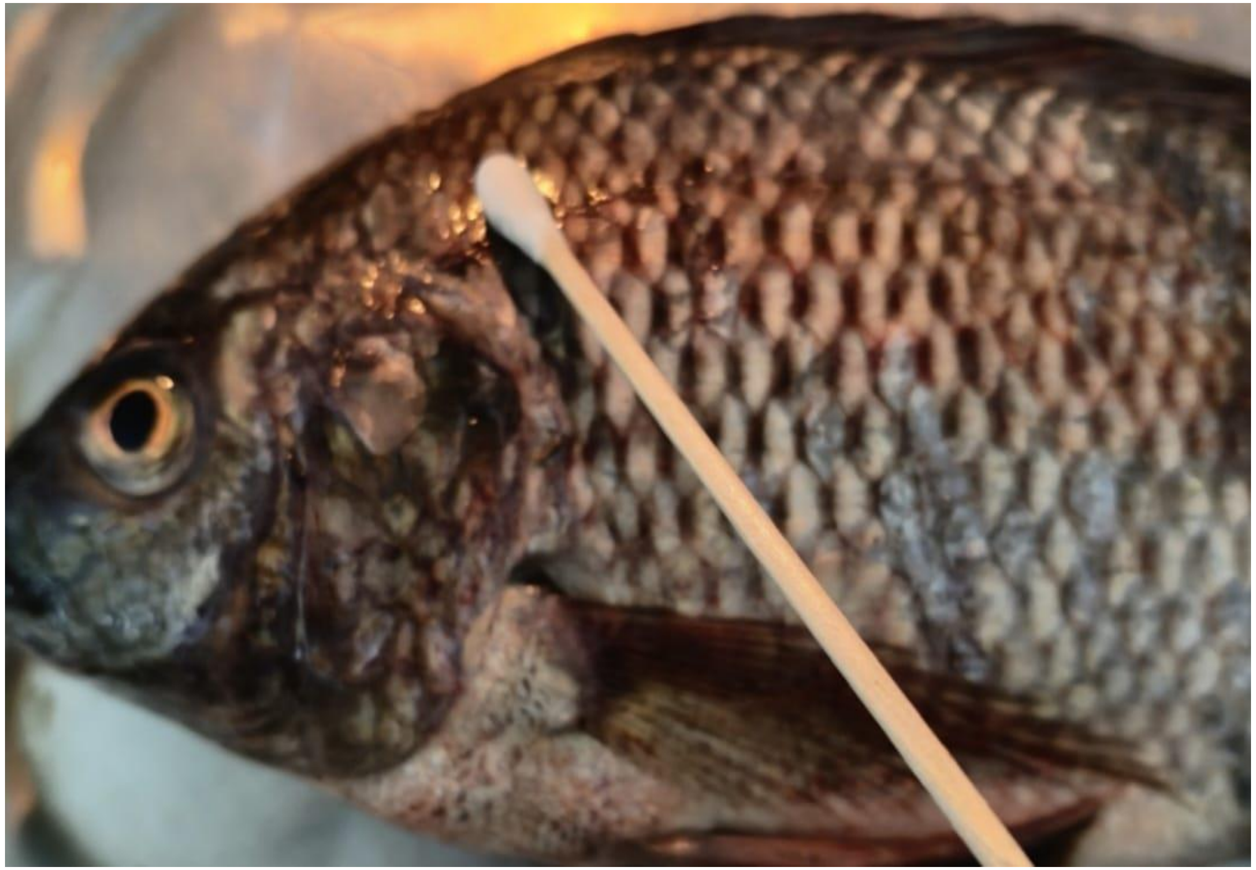

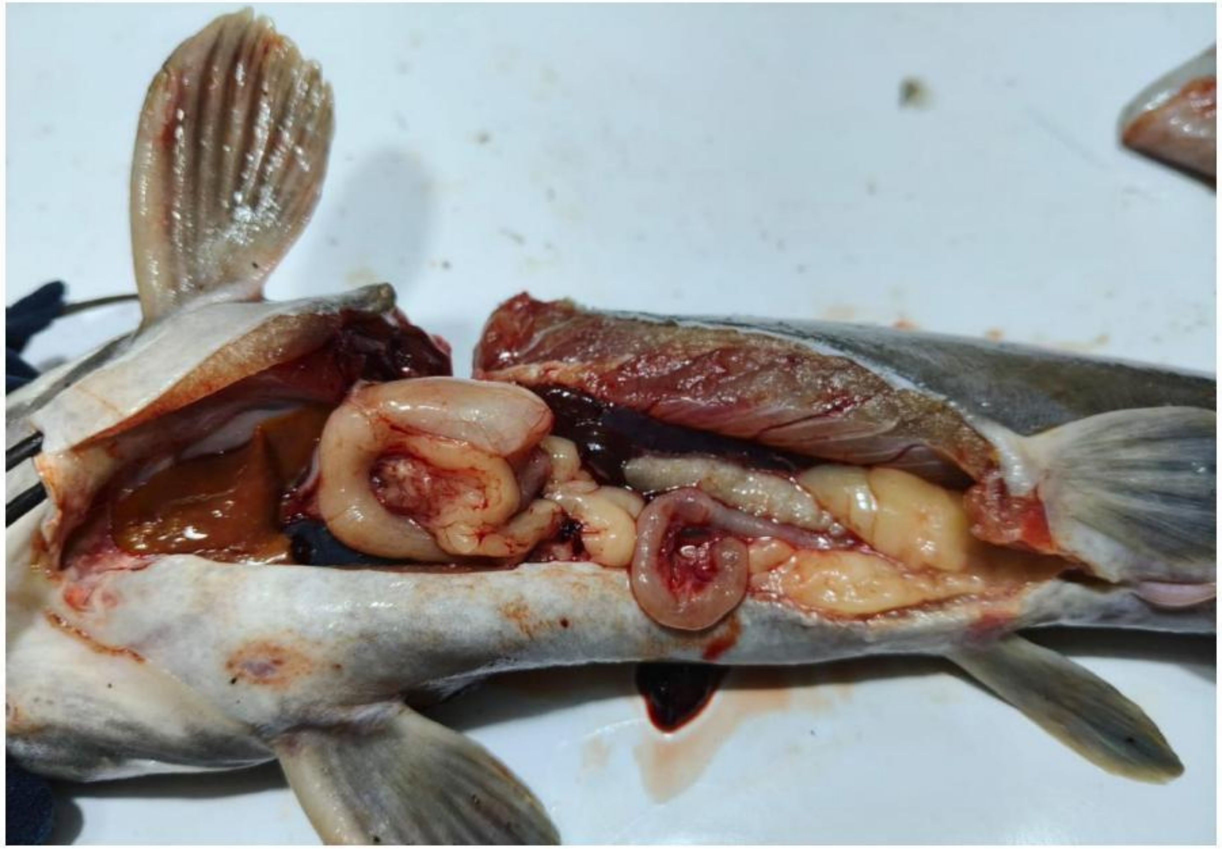

(d) Samples Collection.
