# Supplementary material for: Prevalence and genetic characterization of methicillin-resistant Staphylococcus aureus in Commercial aquaculture farms in Egypt
Source: Sci Rep. 2026 Apr 10;16:12026. doi: 10.1038/s41598-026-40144-y (PMC13068896; doi:10.1038/s41598-026-40144-y)

Supplementary Figure S3:
High-resolution images showing the typical colony characteristics of *S. aureus* on the selective media used

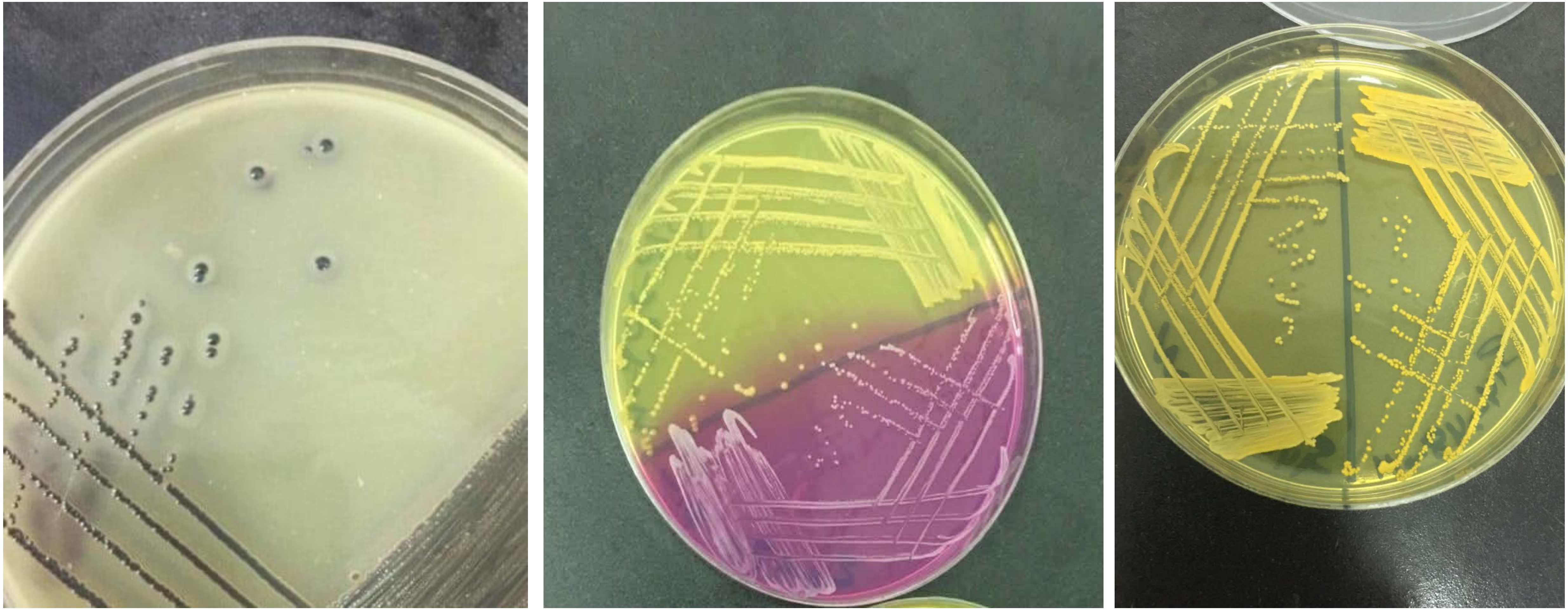

Supplement: Supplementary file 4 — Supplementary Information 3. [file 41598_2026_40144_MOESM4_ESM.pdf]
