# Supplementary material for: Prevalence and genetic characterization of methicillin-resistant Staphylococcus aureus in Commercial aquaculture farms in Egypt
Source: Sci Rep. 2026 Apr 10;16:12026. doi: 10.1038/s41598-026-40144-y (PMC13068896; doi:10.1038/s41598-026-40144-y)

**Supplemental Figure 4:**
**Visual results of the biochemical assays confirming the identification of the isolates.**

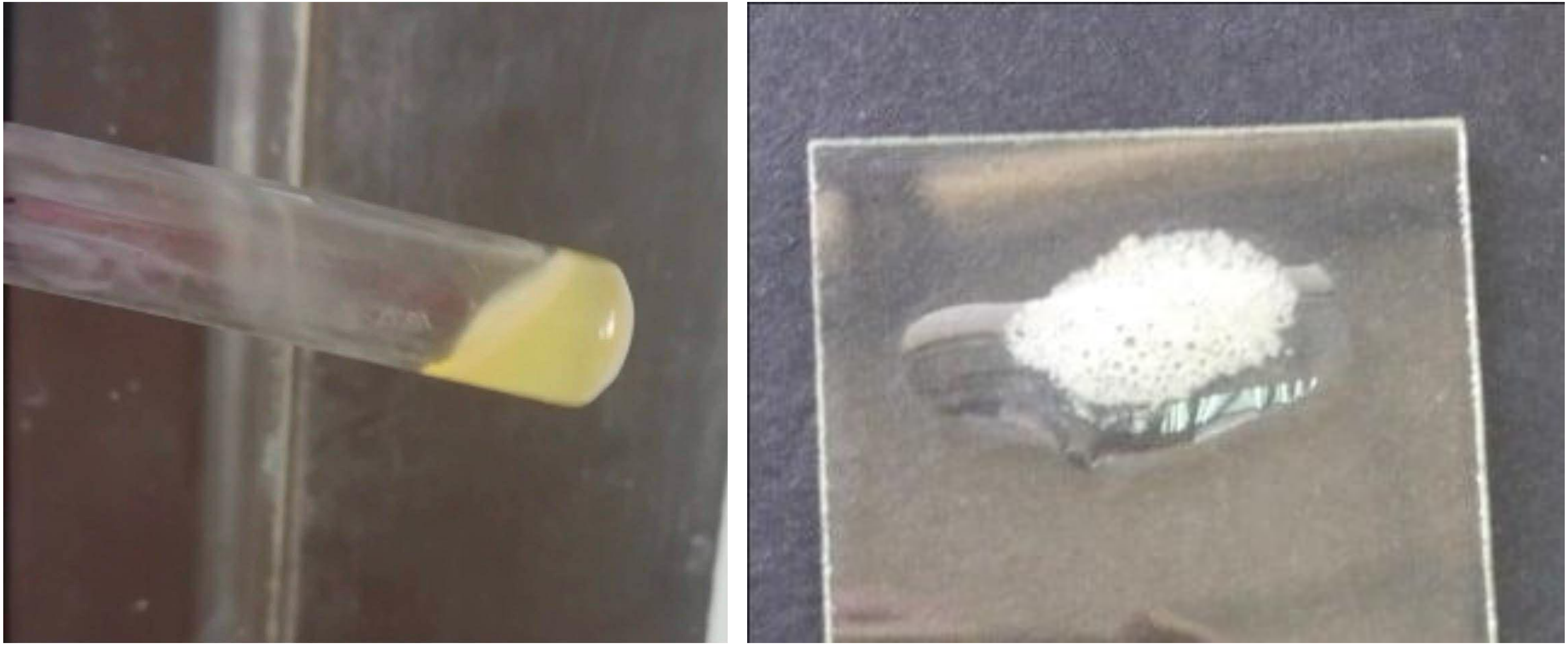

Supplement: Supplementary file 5 — Supplementary Information 4. [file 41598_2026_40144_MOESM5_ESM.pdf]
