# Supplementary material for: Prevalence and genetic characterization of methicillin-resistant Staphylococcus aureus in Commercial aquaculture farms in Egypt
Source: Sci Rep. 2026 Apr 10;16:12026. doi: 10.1038/s41598-026-40144-y (PMC13068896; doi:10.1038/s41598-026-40144-y)
Supplement: Supplementary file 7 — Supplementary Information 6. [file 41598_2026_40144_MOESM7_ESM.doc]

# SUPPLEMENTAL FILE: Target genes, primers and probes

| **Nr**. | **Symbol** | **Synonymes** | **Alleles** | **Name / description of gene or gene product** | **probe** | **Probe sequence** | **Primer sequence** |
| --- | --- | --- | --- | --- | --- | --- | --- |
|  | ***aacA-aphD*** | *-* | *-* | bifunctional enzyme Aac/Aph, gentamicin resistance | aacA-aphD_10,4 | AB096217.1[28286:28313] | AB096217.1[28367:28386:r] |
|  | ***aadD*** | *-* | *-* | aminoglycoside adenyltransferase,  tobramycin resistance | aadD_1,2_PM4 | AB037420.1[788:815] | AB037420.1[854:874:r] |
|  | ***agrB*** | *-* | *agrB-I* | accessory gene regulator B | agrB-I_11 | AF026120.1[3:29] | AF026120.1[57:79:r] |
|  |  | *-* | *agrB-II* |  | agrB-II_11 | AB043554.1[824:852] | AB043554.1[853:871:r] |
|  |  | *-* | *agrB-III* |  | agrB-III_11 | AB043555.1[971:1000] | AB043555.1[1029:1051:r] |
|  |  | *-* | *agrB-IV* |  | agrB-IV_11 | AF288215.1[1200:1226] | AF288215.1[1255:1275:r] |
|  | ***agrC*** | *-* | *agrC-I* | accessory gene regulator C | agrC-I | AB043554.1[1741:1767] | AB043554.1[1826:1846:r] |
|  |  | *-* | *agrC-II* |  | agrC-II | AF001782.1[892:917] | AF001782.1[983:1005:r] |
|  |  | *-* | *agrC-III* |  | agrC-III | AF001783.1[1051:1077] | AF001783.1[1086:1107:r] |
|  |  | *-* | *agrC-IV* |  | agrC-IV | AF288215.1[1553:1580] | AF288215.1[2049:2069:r] |
|  | ***agrD*** | *-* | *agrD-I* | accessory gene regulator D | agrD-I_11  agrD-I_12  agrD-I_13 | AF026120.1[144:171]  AJ617706.1[599:626]  AF210055.1[599:625] | AF026120.1[175:196:r] |
|  |  | *-* | *agrD-II* |  | agrD-II | AF001782.1[650:679] | AF001782.1[681:702:r] |
|  |  | *-* | *agrD-III* |  | agrD-III | AB043555.1[1072:1101] | AB043555.1[1108:1124:r] |
|  | ***aphA3*** | *-* | *-* | 3'5'-aminoglycoside phosphotransferase,  neo-/kanamycin resistance | aphA-3_18,3 | U51474.1[1553:1578:r] | U51474.1[1654:1671] |
|  | ***arcA*** | *-* | *arcA-SCC* | ACME-locus | hp_arcA_611 | AE015929.1[102505:102530:r] | AE015929.1[102460:102479] |
|  | ***arcB*** | *-* | *arcB-SCC* | ACME-locus: ornithincarbamoyltransferase | hp_arcB_611 | AE015929.1[99281:99307:r] | AE015929.1[99256:99274] |
|  | ***arcC*** | *-* | *arcC-SCC* | ACME-locus: carbamatkinase | hp_arcC_611 | AE015929.1[98603:98631:r] | AE015929.1[98571:98590] |
|  | ***arcD*** | *-* | *arcD-SCC* | ACME-locus: arginine/ornithine-antiporter | hp_arcD_611 | AE015929.1[101412:101440:r] | AE015929.1[101381:101398] |
|  | ***aur*** | *-* | *-* | aureolysin | hp_aur_613, hp_aur_611 | AC027136.7[28379:28408:r], AC027136.7[28846:28874:r] | AC027136.7[28345:28365], AC027136.7[28812:28831] |
|  |  | *-* | *aur-MRSA252* |  | hp_aur_613, hp_aur_612 | AC027136.7[28379:28408:r], AJ249166.1[1026:1053] | AC027136.7[28345:28365], AJ249166.1[1067:1088:r] |
|  | ***bap*** | *-* | *-* | surface protein involved in biofilm formation | hp_bap_611 | AY220730.1[7832:7860] | AY220730.1[7869:7891:r] |
|  | ***bbp*** | *-* | *bbp-COL* | bone sialoprotein-binding protein | hp_bbp_616, hp_bbp_614 | AJ005647.1[2220:2246], AB246401.1[76:104] | AB246401.1[2256:2278:r], AB246401.1[132:149:r] |
|  |  | *-* | *bbp-H6606* |  | hp_bbp_611 | AM076252.1[3:31] | AM076252.1[37:59:r] |
|  |  | *-* | *bbp-MRSA252* |  | hp_bbp_613, hp_bbp_614 | AB246401.1[1671:1696], AB246401.1[76:104] | AB246401.1[2373:2393:r], AB246401.1[2256:2278:r], AB246401.1[1720:1739:r], AB246401.1[132:149:r] |
|  |  | *-* | *bbp-MW2* |  | hp_bbp_616, hp_bbp_614 | AJ005647.1[2220:2246], AB246401.1[76:104] | AB246401.1[2373:2393:r], AB246401.1[132:149:r] |
|  |  | *-* | *bbp-Mu50* |  | hp_bbp_617, hp_bbp_614 | AM076243.1[295:323], AB246401.1[76:104] | AB246401.1[2256:2278:r], AB246401.1[132:149:r], AM076243.1[349:368:r] |
|  |  | *-* | *bbp-RF122* |  | hp_bbp_612, hp_bbp_614 | AJ938182.1[578264:578291], AB246401.1[76:104] | AB246401.1[2373:2393:r], AB246401.1[132:149:r], AJ938182.1[578305:578327:r] |
|  | ***blaI*** | *-* | *-* | beta lactamase repressor (inhibitor) | hp_blaI_611 | AB179623.1[12885:12911] | AB179623.1[12931:12952:r] |
|  | ***blaR*** | *-* | *blaR-MRSA252* | beta-lactamase regulatory protein | hp_blaR_612, hp_blaR_611 | AB179623.1[12517:12546], AB179623.1[11832:11859] | AB179623.1[11892:11912:r], AB179623.1[12560:12583:r] |
|  |  | *-* | *blaR-Sepi* |  | hp_blaR_613, hp_blaR_611 | AE015929.1[1662669:1662697:r], AB179623.1[11832:11859] | AE015929.1[1663304:1663323], AB179623.1[12560:12583:r] |
|  | ***blaZ*** | *-* | *-* | beta-lactamase | hp_blaZ_611, blaZ_11 | AB179623.1[10303:10331:r], AB179623.1[10813:10837:r] | AB179623.1[10264:10281], AB179623.1[10713:10735] |
|  | ***blaZ - SCCmec XI*** |  |  | beta-lactamase from SCC*mec* XI | hp_01_blaZ_M10 | FR823292.1[1453:1479:r] | FR823292.1[1429:1448] |
|  | ***capH*** | *-* | *capH1* | capsular polysaccharide synthesis enzyme CapH of capsule types 1, 5, and 8 | hp_capH1_611 | U10927.2[19165:19192] | U10927.2[19210:19230:r] |
|  |  | *-* | *capH5* |  | hp_capH5_611 | AC069081.4[6478:6502] | AC069081.4[6518:6538:r] |
|  |  | *-* | *capH8* |  | hp_capH8_611 | AJ938182.1[120208:120236] | AJ938182.1[120239:120260:r] |
|  | ***capI*** | *-* | *capI8* | capsular polysaccharide biosynthesis protein CapI | hp_capI8_612 | AJ938182.1[121964:121993] | AJ938182.1[122007:122027:r] |
|  | ***capJ*** | *-* | *capJ1* | O-antigen polymerase CapJ of capsule types 1, 5, and 8 | hp_capJ1_611 | U10927.2[21322:21350] | U10927.2[21367:21385:r] |
|  |  | *-* | *capJ5* |  | hp_capJ5_612, hp_capJ5_611 | AC069081.4[8701:8730:r], AC069081.4[8535:8563] | AC069081.4[8673:8694], AC069081.4[8573:8590:r] |
|  |  | *-* | *capJ8* |  | hp_capJ8_611 | AJ938182.1[122465:122495] | AJ938182.1[122504:122521:r] |
|  | ***capK*** | *-* | *capK1* | capsular polysaccharide biosynthesis protein CapK of capsule types 1, 5, and 8 | hp_capK1_611 | U10927.2[22439:22466] | U10927.2[22490:22508:r] |
|  |  | *-* | *capK5* |  | hp_capK5_611 | AC069081.4[9893:9921] | AC069081.4[9933:9952:r] |
|  |  | *-* | *capK8* |  | hp_capK8_611, hp_capK8_612 | AJ938182.1[122685:122712], AJ938182.1[123625:123651] | AJ938182.1[123653:123675:r], AJ938182.1[122716:122740:r] |
|  | ***cat*** | *-* | *cat-pC221* | chloramphenicol acetyltransferase | hp_cat_613 | M64281.1[358:389] | M64281.1[403:426:r] |
|  |  | *-* | *cat-pC223* |  | hp_cat_611 | AF507977.1[17615:17642] | AF507977.1[17658:17682:r] |
|  |  | *-* | *cat-pMC524* |  | hp_cat_612 | AB080798.1[2826:2854] | AJ312056.2[587:605:r], AB080798.1[2860:2878:r] |
|  |  | *-* | *cat-pSBK203R* |  | hp_cat_615 | M58515.1[353:384] | M58515.1[407:431:r] |
|  | ***ccrA*** | *-* | *ccrA-1* | cassette chromosome recombinase A, | hp_ccrA-1_611, hp_ccrA-1_612 | AB033763.2[24367:24393], AB033763.2[24614:24641] | AB033763.2[24397:24416:r], AB033763.2[24649:24667:r] |
|  |  | *-* | *ccrA-2* |  | hp_ccrA-2_612, hp_ccrA-2_611 | AB063172.2[10457:10485], AB063172.2[9903:9931] | AB063172.2[9939:9955:r], AB063172.2[10489:10507:r] |
|  |  | *-* | *ccrA-3* |  | hp_ccrA-3_612, hp_ccrA-3_611 | AB014436.1[787:813], AB014436.1[254:279] | AB014436.1[283:300:r], AB014436.1[826:844:r] |
|  |  | *-* | *ccrA-4* |  | hp_ccrA-4_611 | AF411935.1[8536:8563] | AF411935.1[8803:8823:r] |
|  | ***“ccrAA”*** | *-* | *ccrAA-85-2082* | hypothetical protein accompanying *ccrC* | hp_ccrAA_611 | AB037671.1[61431:61460:r] | AB037671.1[61397:61418] |
|  |  | *-* | *ccrAA-MRSAZH47* |  | hp_ccrAA_613, hp_ccrAA_612 | AM292304.1[6991:7016], AM292304.1[6601:6626] | AM292304.1[6629:6648:r], AM292304.1[7020:7038:r] |
|  | ***ccrB*** | *-* | *ccrB-1* | cassette chromosome recombinase B | hp_ccrB-1_612, hp_ccrB-1_613, hp_ccrB-1_611 | AB033763.2[25387:25411], AB033763.2[25216:25245], AB063171.1[13751:13773] | AB033763.2[25251:25268:r], AB033763.2[25425:25445:r] |
|  |  | *-* | *ccrB-2* |  | hp_ccrB-2_611, hp_ccrB-2_612 | AB063172.2[12275:12302], AB063172.2[12499:12528] | AB063172.2[12548:12567:r], AB096217.1[15001:15022:r], AB063172.2[12308:12331:r] |
|  |  | *-* | *ccrB-3* |  | hp_ccrB-3_611, hp_ccrB-3_612 | AB014436.1[2110:2135], AB014436.1[2215:2243] | AB014436.1[2265:2286:r], AB014436.1[2160:2179:r] |
|  |  | *-* | *ccrB-4* |  | hp_ccrB-4_611 | AE015929.1[59605:59629:r] | AE015929.1[59457:59474] |
|  | ***ccrC*** | *-* | *-* | cassette chromosome recombinase B, | hp_ccrC_611 | AB037671.1[60667:60697:r] | AB037671.1[60643:60662] |
|  | ***cfr*** | *-* | *-* | 23S rRNA methyltransferase | hp_cfr_611 | AJ249217.1[1048:1074] | AJ249217.1[1075:1093:r] |
|  | ***chp*** | *-* | *-* | chemotaxis-inhibiting protein (CHIPS) | hp_chp_611, hp_chp_612 | AB033232.1[8395:8421:r], AB033232.1[8142:8170:r] | AB033232.1[8106:8129], AB033232.1[8353:8373] |
|  | ***clfA*** | *-* | *clfA-COL* | clumping factor A | hp_clfA_612, hp_clfA_611 | AC074317.5[4999:5027:r], AB245456.1[496:524] | AB245456.1[1578:1599:r], AB245456.1[550:569:r] |
|  |  |  | *clfA-MRSA252* |  | hp_clfA_613, hp_clfA_611 | AB245456.1[1551:1576], AB245456.1[496:524] | AB245456.1[1578:1599:r], AB245456.1[550:569:r] |
|  |  |  | *clfA-MW2* |  | hp_clfA_611, hp_clfA_614 | AB245456.1[496:524], AM075836.1[1128:1152] | AB245456.1[1578:1599:r], AB245456.1[550:569:r] |
|  | ***clfB*** | *-* | *clfB-COL* | clumping factor B | hp_clfB_611, hp_clfB_612 | AC027136.7[20622:20652:r], AC027136.7[19674:19705:r] | AC027136.7[20592:20610], AC027136.7[19626:19648] |
|  |  |  | *clfB-MRSA252* |  | hp_clfB_611, hp_clfB_613 | AC027136.7[20622:20652:r], AM075901.1[1069:1098] | AJ938182.1[2647686:2647709], AC027136.7[20592:20610] |
|  |  |  | *clfB-RF122* |  | hp_clfB_614, hp_clfB_611 | AJ938182.1[2647736:2647765:r], AC027136.7[20622:20652:r] | AJ938182.1[2647686:2647709], AC027136.7[20592:20610] |
|  | ***cna*** | *-* | *-* | collagen-binding adhesin | hp_cna_611 | AB266874.1[435:461] | AB266874.1[469:487:r] |
|  | ***coa*** | *-* | *-* | coagulase | hp_coa-105720_PM3 | AB158549.1[3730:3759] | AB158549.1[3772:3793:r] |
|  | ***corB*** | *hl* | *-* | putative membrane protein | hl_11 | BA000017.4 [963137:963165] | BA000017.4[963188:963206:r] |
|  | ***dcs-Q9XB68*** | *-* | *Q9XB68-dcs* | hypothetical protein from SCCmec elements | hp_Q9XB68_611 | AB033763.2[38065:38093:r] | AB033763.2[38014:38037] |
|  | ***dfrA*** | *-* | *-* | dihydrofolate reductase type 1 | hp_dfrA-71304_PM1, hp_dfrA-118680_PM5, hp_dfrA-118679_PM5, hp_dfrA-71303_PM1 | AB049452.1[2172:2198], AB049452.1[2179:2205], AB049452.1[2096:2120:r], AB049452.1[2076:2103] | AB049452.1[2280:2299:r] |
|  | ***ebh*** | *-* | *-* | cell wall associated fibronectin-binding protein | hp_ebh-3prime_611 | AJ938182.1[1406914:1406940:r] | AJ938182.1[1405560:1405579] |
|  | ***ebpS*** | *-* | *ebpS* | cell surface elastin binding protein | hp_ebpS_614, hp_ebpS_612, hp_ebpS_613 | AF400161.1[975:999], AF400161.1[273:300], AF400161.1[511:536] | AF400161.1[1013:1030:r], AF400161.1[546:569:r], AF400161.1[303:321:r] |
|  |  |  | *ebpS-01-1111* (aus CC45) |  | hp_ebpS_614, hp_ebpS_611 | AF400161.1[975:999], AM075954.1[148:172] | AF400161.1[1013:1030:r], AF400161.1[303:321:r] |
|  | ***edinA*** | *-* | *-* | epidermal cell differentiation inhibitor precursor | edinA_11 | M63917.1[460:489] | M63917.1[502:520:r] |
|  | ***edinB*** | *-* | *-* | epidermal cell differentiation inhibitor B | edinB_11 | AB057421.1[7445:7471] | AB057421.1[7482:7501:r] |
|  | ***edinC*** | *-* | *-* | epidermal cell differentiation inhibitor C | edinC_11 | AP003088.1[1810:1839:r] | AP003088.1[1755:1776] |
|  | ***eno*** | *-* | *-* | enolase | hp_eno_611 | AC074317.5[16708:16737:r] | AC074317.5[16665:16683] |
|  | ***“entX”*** | SACOL1657 | *-* | hypothetical enterotoxin homologue | entX_11 | BA000017.4[1708858:1708886:r] | BA000017.4[1708835:1708855] |
|  | ***erm*(A)** | ***ermA*** | *-* | rRNA adenine N-6-methyltransferase, erythromycin/ clindamycin resistance | hp_ermA-59239_PM1 | AB037671.1[52962:52987] | AB037671.1[53019:53040:r] |
|  | ***erm*(B)** | ***ermB*** | *-* | erythromycin/clindamycin resistance | hp_ermB_611  hp_ermB_612 | EF450709.1 [2405:2428]  EF450709.1 [2528:2557] | EF450709.1 [2443:2464:r]  EF450709.1 [2564:2584:r] |
|  | ***erm*(C)** | ***ermC*** | *-* | erythromycin/clindamycin resistance | ermC_8,1_PM4 ermC_8,2_PM4 | AE002098.2[75789:75813], AE002098.2[75854:75878] | AE002098.2[75909:75929:r] |
|  | ***etA*** | *-* | *-* | exfoliative toxin serotype A | 8,2-etA | AP001553.1[42317:42344] | AP001553.1[42387:42406:r] |
|  | ***etB*** | *-* | *-* | exfoliative toxin serotype B | 9,3-etB | AP003088.1[5389:5416] | AP003088.1[5438:5460:r] |
|  | ***etD*** | *-* | *-* | exfoliative toxin D | etD_11 | AB057421.1[5648:5677] | AB057421.1[5694:5715:r] |
|  | ***far1*** | ***fusB*** | *-* | fusidic acid resistance (plasmid-borne) | far1_10 | AY047358.1 [1787:1814] | AY047358.1[1818:1838:r] |
|  | ***fexA*** | *-* | *-* | chloramphenicol/florfenicol exporter | hp_fexA_611 | AJ549214.1[332:357] | AJ549214.1[364:382:r] |
|  | ***fib*** | *-* | *-* | fibrinogen binding protein (19 kDa) | hp_fib_611, hp_fib_612 | AJ306909.1[483:507], BX571856.1[1178081:1178105] | AJ306909.1[511:528:r] |
|  | ***fnbA*** | *-* | *fnbA-COL* | fibronectin-binding protein A | hp_fnbA_615, hp_fnbA_612 | AJ629121.1[1767:1795], AJ629121.1[983:1009] | AJ629121.1[1823:1843:r], AJ629121.1[1031:1052:r] |
|  |  |  | *fnbA-MRSA252* |  | hp_fnbA_615, hp_fnbA_613 | AJ629121.1[1767:1795], AM076028.1[1027:1055] | AM076028.1[1061:1079:r], AJ629121.1[1823:1843:r] |
|  |  |  | *fnbA-MW2* |  | hp_fnbA_615, hp_fnbA_611 | AJ629121.1[1767:1795], AM075994.1[630:657] | AM075994.1[672:693:r], AJ629121.1[1823:1843:r] |
|  |  |  | *fnbA-RF122* |  | hp_fnbA_615, hp_fnbA_614 | AJ629121.1[1767:1795], AJ938182.1[2510055:2510084:r] | AJ629121.1[1823:1843:r], AJ938182.1[2510030:2510048] |
|  |  |  | *fnbA-ST80* |  | hp_fnbA_615 | AJ629121.1[1767:1795] | AJ629121.1[1823:1843:r] |
|  | ***fnbB*** | *-* | *fnbB-COL* | fibronectin-binding protein B | hp_fnbB_614, hp_fnbB_616 | AJ629122.1[991:1017], AJ629122.1[1658:1688] | AJ629122.1[1041:1061:r], AJ629122.1[1697:1714:r] |
|  |  |  | *fnbB-MW2* |  | hp_fnbB_613, hp_fnbB_616 | AM076068.1[854:883], AJ629122.1[1658:1688] | AM076068.1[893:912:r] |
|  |  |  | *fnbB-Mu50* |  | hp_fnbB_611, hp_fnbB_616 | AM076047.1[866:895], AJ629122.1[1658:1688] | AM076047.1[795:817:r], AJ629122.1[1697:1714:r], AM076047.1[914:934:r] |
|  |  |  | *fnbB-ST15* |  | hp_fnbB_611, hp_fnbB_612 | AM076047.1[866:895], AM076087.1[758:783] | AM076057.1[920:940:r], AM076047.1[795:817:r] |
|  |  |  | *fnbB-ST36* |  | hp_fnbB_611 | AM076047.1[866:895] | AM076047.1[795:817:r], AM076079.1[914:933:r] |
|  |  |  | *fnbB-ST45-2* |  | hp_fnbB_615 | AM076078.1[866:893] | AM076047.1[795:817:r], AM076078.1[900:920:r] |
|  | ***fosB*** | *-* | *fosB* | metallothiol transferase | hp_fosB_611 | AP009324.1[2460791:2460821] | AP009324.1[2460852:2460871:r] |
|  |  |  | *fosB-plasmid* |  | hp_fosB_612 | AP006717.1[448:478] | AP006717.1[508:527:r] |
|  | ***fusC*** | **Q6GD50** | *-* | SCC-encoded protein associated with fusidic acid resistance | hp_Q6GD50_611 | AF411935.1[423:452:r] | AF411935.1[372:390] |
|  | ***gapA*** | *-* | *-* | glyceraldehyde 3-phosphate dehydrogenase, locus 1 | hp_gapA-118704_PM5 | AC074317.5[21403:21431:r] | AC074317.5[21373:21393] |
|  | ***hla*** | *-* | *-* | haemolysin alpha | hla_11 | AC027137.8[47755:47784] | AC027137.8[47798:47820:r] |
|  | ***hlb*** | *-* | *-* | haemolysin beta | hp_hlb_611, hp_hlb_612, hp_hlb-118707_PM5, hp_hlb_613, hp_hlb-118708_PM5 | AJ938182.1[2010526:2010550], AP009324.1[2127638:2127663], AP009324.1[2084497:2084523], S72497.1[366:390], S72497.1[347:374] | AJ938182.1[2010553:2010572:r] |
|  | ***hld*** | *-* | *-* | haemolysin delta | hld_11 | AB043554.1[196:220:r] | AB043554.1[153:175] |
|  | ***hlgA*** | *-* | *-* | haemolysin gamma, component A | hlgA_11 | BA000017.4 [2549687:2549713] | BA000017.4[2549714:2549735:r] |
|  | ***hlIII*** | SAV2170, SACOL2160 | *hlIII- other than RF122* | putative membrane protein | hl-III_11 | AB078343.1[539:567] | AB078343.1[998:1019:r] |
|  |  |  | *hlIII-consensus* |  | hp_hlIII_611 | AB078343.1[951:976] | AB078343.1[590:612:r] |
|  | ***hsdS1*** | *-* | *hsdS1-RF122* | type I site-specific deoxyribonuclease subunit, 1^st^ locus | hp_hsdS-RF122-1_611 | AJ938182.1[317663:317689] | AJ938182.1[317702:317723:r] |
|  | ***hsdS2*** | *-* | *hsdS2-ST5+ST8* | type I site-specific deoxyribonuclease subunit, 2nd locus | hp_hsdS-COL-1_611 | AP009324.1[478662:478690] | AP009324.1[478701:478719:r] |
|  |  |  | *hsdS2-MW2+476* |  | hp_hsdS-MW2-1_611 | BA000033.2[442980:443006] | BA000033.2[443035:443058:r], AJ938182.1[1812895:1812912] |
|  |  |  | *hsdS2-RF122* |  | hp_hsdS-RF122-2_611 | AJ938182.1[422326:422351] | AJ938182.1[422376:422398:r] |
|  |  |  | *hsdS2-MRSA252* |  | hp_hsdS-MRSA252-1_611 | BX571856.1[463045:463073] | BX571856.1[463099:463120:r] |
|  | ***hsdS3*** | *-* | *hsdS3-AllOtherThanRF122+252* | type I site-specific deoxyribonuclease subunit, 3rd locus | hp_hsdS-CC25_611 | DQ309452.1[57:85] | AJ938182.1[1812895:1812912] |
|  |  |  | *hsdS3-ST8+ST1+RF122* |  | hp_hsdS-COL-2_611 | AJ938182.1[1811960:1811988:r] | AJ938182.1[1812895:1812912] |
|  |  |  | *hsdS3-Mu50+N315* |  | hp_hsdS-Mu50-2_611 | AP009324.1[1937287:1937313:r] | AP009324.1[1937243:1937264] |
|  |  |  | *hsdS3-CC51+252* |  | hp_hsdS-CC51_611 | BX571856.1[1983689:1983715:r] | BX571856.1[1983667:1983686] |
|  |  |  | *hsdS3-MRSA252* |  | hp_hsdS-MRSA252-2_611 | BX571856.1[1983034:1983063:r] | BX571856.1[1983667:1983686] |
|  | ***hsdSx*** | *-* | *hsdSx-CC25* | type I site-specific deoxyribonuclease subunit, unknown locus | hp_hsdS-CC25_612 | AP009324.1[1938268:1938295:r] | AJ938182.1[1811926:1811943] |
|  |  |  | *hsdSx-CC15* |  | hp_hsdS-CC15_611 | DQ309450.1[976:1000] | DQ309450.1[1009:1031:r] |
|  |  |  | *hsdSx-etd* |  | hp_hsdS-etd_611 | AB057421.1[2572:2598:r] | AB057421.1[2543:2565] |
|  | ***hysA1/2*** | *-* | *hysA1-MRSA252* | hyaluronate lyase, first / second locus | hp_hysA_613 | BX571856.1[1975471:1975495], | AC078831.10[18640:18660:r], AJ938182.1[1803841:1803863:r] |
|  |  |  | *hysA1-MRSA252+RF122 and hysA2-all* |  | hp_hysA_614 | AC078831.10[18606:18636] |  |
|  |  |  | *hysA1-MRSA252+RF122 and hysA2-COL+USA300* |  | hp_hysA_615 | AJ938182.1[1803808:1803836] |  |
|  | ***hysA2*** | *-* | *hysA2-AllOtherThan252* | hyaluronate lyase, second locus | hp_hysA_611 | AC078831.10[17303:17329], | AC078831.10[17230:17253:r], AC078831.10[17343:17361:r], AC078831.10[18640:18660:r] |
|  |  |  | *hysA2-COL+USA300+NCTC* |  | hp_hysA_617 | AC078831.10[17198:17228] |  |
|  |  |  | *hysA2-AllOtherThan COL+USA300+NCTC* |  | hp_hysA_616 | AJ938182.1[2230577:2230606] |  |
|  |  |  | *hysA2-OtherThan COL+USA300+NCTC* |  | hp_hysA_618 | AP009324.1[2345045:2345075], |  |
|  |  |  | *hysA2-MRSA252* |  | hp_hysA_612 | AY442448.1[26:54], |  |
|  | ***icaA*** | *-* | *-* | intercellular adhesion protein A | hp_icaA_611 | AF086783.1[3376:3401] | AF086783.1[3422:3442:r] |
|  | ***icaC*** | *-* | *-* | intercellular adhesion protein C | hp_icaC_611 | AF086783.1[5365:5394] | AF086783.1[5402:5420:r] |
|  | ***icaD*** | *-* | *-* | biofilm PIA synthesis protein D | hp_icaD_611 | AF086783.1[3681:3710] | AF086783.1[3738:3760:r] |
|  | ***isaB*** |  | *isaB* | immunodominant antigen B | hp_isaB_611 | AC027136.7[30242:30266:r] | AC027136.7[30213:30231] |
|  |  |  | *isaB-MRSA252* |  | hp_isaB_612 | BX571856.1[2813801:2813828:r] | BX571856.1[2813775:2813793] |
|  | ***isdA*** | *-* | *isdA* | transferrin-binding protein | hp_isdA_611, hp_isdA_612, hp_isdA_614 | AB042826.1[872:896], AB042826.1[991:1015], AJ938182.1[1075980:1076008:r] | AB042826.1[1037:1059:r], AB042826.1[901:919:r], AJ938182.1[1075946:1075968] |
|  |  |  | *isdA-MRSA252* |  | hp_isdA_611, hp_isdA_612 | AB042826.1[872:896], AB042826.1[991:1015] | AB042826.1[1037:1059:r], AB042826.1[901:919:r] |
|  | ***katA*** | *-* | *-* | katalase A | katA_11 | BA000017.4 [1409422:1409446] | BA000017.4[1409453:1409473:r] |
|  | ***kdpA*** | *-* | *kdpA-SCC* | potassium-translocating ATPase A, chain 2 | hp_kdpA-SCC_612, hp_kdpA-SCC_611 | AB033232.1[25:53], AP006716.1[38978:39004] | AB033232.1[63:83:r], AP006716.1[39037:39057:r] |
|  | ***kdpB*** | *-* | *kdpB-SCC* | potassium-transporting ATPase B, chain 1 | hp_kdpB-SCC_611 | AB033232.1[1908:1935] | AB033232.1[1948:1967:r] |
|  | ***kdpC*** | *-* | *kdpC-SCC* | potassium-translocating ATPase C, chain 2 | hp_kdpC-SCC_611, hp_kdpC-SCC_612 | AB033232.1[3099:3125], AB033232.1[3206:3232:r] | AB033232.1[3133:3151:r], AB033232.1[3182:3202] |
|  | ***kdpD*** | *-* | *kdpD-SCC* | sensor kinase protein | hp_kdpD-SCC_611 | AP006716.1[37752:37779:r] | AP006716.1[37713:37731] |
|  | ***kdpE*** | *-* | *kdpE-SCC* | KDP operon transcriptional regulatory protein | hp_kdpE-SCC_611 | AP006716.1[35126:35151:r] | AP006716.1[35099:35117] |
|  | ***linA*** | *-* | *-* | lincosamid-nucleotidyltransferase | linA_19,2  linA_19,3 | AM184101.1[2144:2168:r], AM184101.1[2216:2240:r] | AM184101.1[2037:2057], AM184101.1[2053:2070] |
|  | ***lmrP*** | *-* | *-* | hypothetical protein, similar to integral membrane protein LmrP | hp_lmrP_613, hp_lmrP_611 | AC069081.4[27540:27566], AC069081.4[26853:26878] | AC069081.4[26886:26903:r], AC069081.4[27570:27590:r] |
|  |  |  | *lmrP-RF122* |  | hp_lmrP_612, hp_lmrP_614 | AJ938182.1[140620:140646], AJ938182.1[141308:141333] | AJ938182.1[140655:140672:r], AJ938182.1[141338:141358:r] |
|  | ***lukD*** | *-* | *-* | leukocidin D component | lukD_11 | AB055623.1[2367:2396] | AB055623.1[2421:2441:r] |
|  | ***lukE*** | *-* | *-* | leukocidin E component | lukE_11 | AB055623.1[1159:1183] | AB055623.1[1207:1226:r] |
|  | ***lukF-hlg*** | *-* | *-* | haemolysin gamma, component B | lukF-10 | BA000017.4[2552175:2552200] | BA000017.4[2552204:2552221:r] |
|  | ***lukF-PV*** | *-* | *-* | Panton Valentine leukocidin F component | lukF-PV_10 | AB006796.1[2256:2284] | AB006796.1[2295:2316:r] |
|  | ***lukF-PV83*** | *-* | *-* | F component from hypothetical leukocidin from ruminants | lukF-PV-P83_11 | AB044554.1[42010:42037] | AB044554.1[42053:42070:r] |
|  | ***lukM*** | *-* | *-* | S component from hypothetical leukocidin from ruminants | lukM_11 | AB044554.1[40866:40893] | AB044554.1[40914:40932:r] |
|  | ***lukS-hlg*** | *-* | *lukS* | haemolysin gamma, component C | lukS_10 | BA000017.4 [2551185:2551209] | BA000017.4[2551213:2551233:r] |
|  |  |  | *lukS-ST45* |  | hp_lukS-ST45_611 | EF672356.1[663:686] | EF672356.1[690:710:r]  EF672356.1[690:711:r] |
|  | ***lukS-PV*** | *-* | *-* | Panton Valentine leukocidin S component | lukS-PV_20 | AB006796.1[1628:1656] | AB006796.1[1679:1699:r] |
|  | ***“lukX”*** | SAV2004, *lukG, lukA* | *-* | leukocidin/haemolysin toxin family protein | lukX_11 | BA000017.4[2127329:2127353:r] | BA000017.4[2127284:2127306] |
|  | ***“lukY”*** | SAV2005, *lukH, lukB* | *lukY* | leukocidin/haemolysin toxin family protein | lukY-var1_11 | BA000017.4[2129068:2129097:r] | BA000017.4[2129030:2129050] |
|  |  |  | *lukY-MRSA252* |  | lukY-var2_11 | BX571856.1[2171414:2171443:r] |  |
|  | ***map*** | *eap* | *map-7* | Major histocompatibility complex class II analogue protein (=Extracellular adherence protein, *eap*) | hp_map_613 | AJ132841.1[368:395] | AJ132841.1[420:438:r], AJ223806.1[279:298:r] |
|  |  |  | *map-COL* |  | hp_map_611 | AJ223806.1[226:254] |  |
|  |  |  | *map-IPOP2* |  | hp_map_612 | AJ245439.1[361:385] |  |
|  | ***mecA*** | *-* | *-* | Modified penicillin binding protein 2, beta-lactam resistance defining MRSA | hp_mecA-118720_PM5, hp_mecA-59223_PM1 | AB033763.2[32971:32998], AB033763.2[33097:33124] | AB033763.2[33014:33032:r], AB033763.2[33163:33182:r] |
|  | ***mecC*** |  |  | Alternative *mec* gene from SCC*mec* XI | 17_mecA_hp  25_mecA_hp | FR823292.1[3006:3032:r]  FR823292.1[2830:2862:r] | FR823292.1[2968:2988:r]  FR823292.1[2764:2788:r] |
|  | ***mecI*** | *-* | *-* | methicillin-resistance regulatory protein | hp_mecI_611 | AB037671.1[22423:22452:r] | AB037671.1[22395:22416] |
|  | ***mecR1*** | *mecR* | *-* | signal transducer protein MecR1 | hp_mecR_611, hp_mecR_612 | AB033763.2[31157:31186:r], AB037671.1[22874:22900:r] | AB033763.2[31133:31154], AB037671.1[22840:22859] |
|  | ***mefA*** | *-* | *-* | macrolide efflux protein A | hp_mefA_611, hp_mefA_612 | AB011259.1[536:563], AB011259.1[1045:1072] | AB011259.1[570:588:r], AB011259.1[1078:1099:r] |
|  | ***merA*** | *-* | *-* | mercury-reductase | hp_merA_611 | AB037671.1[39354:39382:r] | AB037671.1[39315:39334] |
|  | ***merB*** | *-* | *-* | mercuric resistance operon regulatory protein | hp_merB_611 | AB037671.1[38021:38046:r] | AB037671.1[38000:38018] |
|  | ***mph*(BM)** | *mpbBM,*  *mphBM* | *-* | probable lysylphosphatidylglycerol synthetase | hp_mpbBM_611, hp_mpbBM_612 | AB013298.1[2664:2693], AB013298.1[2896:2924] | AB013298.1[2700:2720:r], AB013298.1[2929:2947:r] |
|  | ***mprF*** | *-* | *-* | energy-dependent efflux of erythromycin | hp_mprF_612, hp_mprF_611 | AP009324.1[1443571:1443601], AB043507.1[2677:2707] | AB043507.1[2708:2731:r] |
|  | ***msr*(A)** | *msrA* | *-* | mercuric resistance operon regulatory protein | msrA_15,3 | AB013298.1[1525:1552] | AB013298.1[1614:1635:r] |
|  | ***mupR*** | *mupA* | *-* | mupirocin resistance protein | mupR_13,2 | X75439.1[1504:1531] | X75439.1[1623:1642:r] |
|  | ***nuc1*** | *-* | *-* | thermostable extracellular nuclease | hp_nuc1_611 | AJ938182.1[825403:825429] | AJ938182.1[825445:825462:r] |
|  | ***ORF CM14*** | SAB0026 | *-* | enterotoxin-like protein ORF CM14 | hp_entCM14_611, hp_entCM14_612 | AJ938182.1[37154:37182], AJ938182.1[37532:37557] | AJ938182.1[37591:37610:r], AJ938182.1[37203:37221:r] |
|  | ***pls-SCC*** | *-* | *-* | plasmin-sensitive surface protein | hp_plsSCC_611 | AB033763.2[15685:15709:r] | AB033763.2[15641:15661] |
|  | ***Q2FXC0*** | *-* | *-* | hypothetical protein, located next to serine protease operon | hp_Q2FXC0_611 | AP009351.1[1905375:1905400] | AP009351.1[1905410:1905429:r] |
|  | ***Q2YUB3*** | *-* | *-* | unspecific efflux/transporter | hp_Q2YUB3_611 | AJ938182.1[2026944:2026969:r] | AJ938182.1[2026920:2026937] |
|  | ***Q7A4X2*** | *-* | *-* | hypothetical protein | hp_Q7A4X2_611 | AJ938182.1[1837306:1837335] | AJ938182.1[1837354:1837376:r] |
|  | ***qacA*** | *-* | *-* | quaternary ammonium compound resistance protein A | hp_qacA_611 | AB255366.1[19119:19147] | AB255366.1[20504:20526:r], AB255366.1[20475:20498:r], AB255366.1[19153:19173:r] |
|  | ***qacC*** | *-* | *qacC* | quaternary ammonium compound resistance protein C | hp_qacC_611 | AB125342.1[2382:2411] | AB125342.1[2431:2450:r] |
|  |  |  | *qacC-SA5* |  | hp_qacC_613 | U81980.1[2017:2043] | U81980.1[2065:2086:r] |
|  |  |  | *qacC-ST94* |  | hp_qacC_615 | Y16944.1[1622:1649] | Y16944.1[1692:1714:r] |
|  |  |  | *qacC-Ssap* |  | hp_qacC_612 | Y16945.1[1951:1981] | AE016833.1[8848:8869:r] |
|  |  |  | *qacC-equine* |  | hp_qacC_614 | AJ512814.1[1518:1545] | AJ512814.1[1567:1590:r] |
|  | ***rrn STAU*** | *-* | *-* | Ribosomal sequence from *S. aureus* (genus-specific positive control) | s_aur_rrn_1_pm_PM4 | BA000017.4[1999541:1999566:r]  BA000018.3[1921746:1921771:r] | BA000017.4[1999503:1999521:r]  BA000018.3[1921708:1921726:r] |
|  | ***saeR*** | *-* | *-* | response regulator, sae locus | hp_saeR_611 | AF129010.1[251:280] | AF129010.1[296:316:r] |
|  | ***saeS*** | *-* | *-* | histidine protein kinase, sae locus | hp_saeS_611, hp_saeS_612 | AF129010.1[1229:1257], AF129010.1[1729:1758] | AF129010.1[1283:1304:r], AF129010.1[1770:1791:r] |
|  | ***sak*** | *-* | *-* | staphylokinase | hp_sak_611, hp_sak-118723_PM5 | AB033232.1[6502:6531], AB033232.1[6660:6685] | AB033232.1[6532:6550:r], AB033232.1[6708:6727:r] |
|  | ***sarA*** | *-* | *-* | staphylococcal accessory regulator A | hp_sarA_611, hp_sarA-118724_PM5 | AF515775.1[1114:1140], AF515775.1[963:988] | AF515775.1[1150:1167:r], AF515775.1[990:1008:r] |
|  | ***sasG*** | *-* | *sasG-COL* | *Staphylococcus aureus* surface protein G | hp_sasG_613, hp_sasG_611 | AP009324.1[2635299:2635327:r], AP009324.1[2635746:2635773:r] | AP009324.1[2635268:2635289], AP009324.1[2635703:2635725] |
|  |  |  | *sasG-MW2* |  | hp_sasG_612, hp_sasG_611 | BA000033.2[2573572:2573601:r], AP009324.1[2635746:2635773:r] | AP009324.1[2635703:2635725], BA000033.2[2573526:2573543] |
|  | ***sat*** | *-* | *-* | streptothricin-acetyltransferase | sat-17,2  sat-17,3 | U51474.1[393:421]  U51474.1[429:456] | U51474.1[488:505:r] |
|  | ***sbi*** | *-* | *-* | IgG-binding protein | hp_sbi-118725_PM5, hp_sbi-118726_PM5 | AB050860.1[387:412], AB050860.1[625:654] | AB050860.1[446:465:r], AB050860.1[671:693:r] |
|  | ***scn*** | *-* |  | Staphylococcal complement inhibitor (SCIN) | hp_scn_611 | AF424783.1[41412:41440] | AF424783.1[41450:41470:r] |
|  | ***sdrC*** | *-* | *sdrC-B1* | Ser-Asp rich fibrinogen-/bone sialoprotein-binding protein C | hp_sdrC_612, hp_sdrC_613 | AM076155.1[1009:1036], AJ005645.1[679:705] | AJ938182.1[574454:574471:r], AM076155.1[1039:1061:r] |
|  |  |  | *sdrC-COL* |  | hp_sdrC_611, hp_sdrC_613  hp_sdrC_615, | AJ005645.1[280:308], AJ005645.1[679:705]  AJ005645.1[1288:1314], | AJ005645.1[322:341:r], AJ005645.1[726:744:r]  AJ005645.1[1337:1355:r], |
|  |  |  | *sdrC-MRSA252* |  | hp_sdrC_616, hp_sdrC_613 | AJ938182.1[575014:575042], AJ005645.1[679:705] | AJ938182.1[574454:574471:r] |
|  |  |  | *sdrC-MW2* |  | hp_sdrC_616, hp_sdrC_611, hp_sdrC_613 | AJ938182.1[575014:575042], AJ005645.1[280:308], AJ005645.1[679:705] | AJ005645.1[322:341:r], AJ005645.1[1337:1355:r], AJ005645.1[726:744:r] |
|  |  |  | *sdrC-Mu50* |  | hp_sdrC_614, hp_sdrC_611, hp_sdrC_613 | AM076143.1[1099:1125], AJ005645.1[280:308], AJ005645.1[679:705] | AJ005645.1[322:341:r], AJ005645.1[1337:1355:r], AJ005645.1[726:744:r] |
|  | ***sdrD*** | *-* | *sdrD-COL* | Ser-Asp rich fibrinogen-/bone sialoprotein-binding protein D | hp_sdrD_614, hp_sdrD_612 | AJ005646.1[1866:1893], AJ005646.1[1207:1232] | AJ005646.1[1237:1258:r], AJ005646.1[1903:1924:r] |
|  |  |  | *sdrD-Mu50* |  | hp_sdrD_614, hp_sdrD_613 | AJ005646.1[1866:1893], AM076196.1[158:186] | AJ005646.1[1903:1924:r], AM076196.1[190:210:r] |
|  |  |  | *sdrD-other1* |  | hp_sdrD_614, hp_sdrD_611 | AJ005646.1[1866:1893], AM076206.1[157:186] | AJ005646.1[1903:1924:r], AM076206.1[195:212:r] |
|  | ***sdrM*** | *tet*Efflux | *-* | transport-/efflux protein | hp_tetEfflux_611 | AB078343.1[1808:1834] | AB078343.1[1849:1870:r] |
|  | ***sea*** | *entA* | *sea* | enterotoxin A | hp_entA-71320_PM1, hp_entA-118684_PM5, hp_entA-71321_PM1 | AP009324.1[2089969:2089995:r], AP009324.1[2089909:2089933:r], AP009324.1[2089853:2089879:r] | AP009324.1[2089870:2089889] |
|  |  |  | *sea-320E (entA-320E)* | enterotoxin A, allele from strain 320E | entA-var2_11 | AY196686.1\|[508:532] |  |
|  |  | *entP, sep* | *sea-N315* | enterotoxin A, allele from strain N315 =enterotoxin P | entA-var3_11 | BA000018.3[2011518:2011545:r] | BA000018.3[2011492:2011510] |
|  | ***seb*** | *entB* | *-* | enterotoxin B | hp_entB-71322_PM3, hp_entB-118687_PM5 | AF410775.1[2675:2701:r], AF410775.1[2357:2381:r] | AF410775.1[2621:2642], AF410775.1[2308:2327] |
|  | ***sec*** | *entC* | *-* | enterotoxin C | hp_entC-71327_PM1, hp_entC-71326_PM1 | AB084256.1[611:639], AB084256.1[530:558] | AB084256.1[681:698:r] |
|  | ***sed*** | *entD* | *-* | enterotoxin D | entD_11 | AY518388.1[49:76] | AY518388.1[79:97:r] |
|  | ***see*** | *entE* | *-* | enterotoxin E | entE_11 | M21319.1[601:624] | M21319.1[644:661:r] |
|  | ***seg*** | *entG* | *-* | enterotoxin G | entG_11 | BA000017.4 [1954500:1954526:r] | BA000017.4[1954468:1954486] |
|  | ***seh*** | *entH* | *-* | enterotoxin H | hp_entH-118692_PM5 | AB060536.1[139:164] | AB060536.1[178:196:r] |
|  | ***sei*** | *entI* | *-* | enterotoxin I | entG_11 | BA000017.4[1957319:1957343:r] | BA000017.4[1957273:1957293] |
|  | ***sej*** | *entJ* | *-* | enterotoxin J | hp_entJ-118694_PM5 | AB075606.1[1849:1876:r] | AB075606.1[1804:1823] |
|  | ***sek*** | *entK* | *-* | enterotoxin K | hp_entK_612, hp_entK_611 | AF410775.1[14220:14250], AF410775.1[13901:13932] | AF410775.1[14306:14328:r], AF410775.1[14268:14290:r], AF410775.1[13951:13974:r] |
|  | ***sel*** | *entL* | *-* | enterotoxin L | entL_11 | AF217235.1[892:919] | AF217235.1[930:950:r] |
|  | ***sem*** | *entM* | *-* | enterotoxin M | entM_11 | BA000017.4[1958262:1958291:r] | BA000017.4[1958242:1958260]] |
|  | ***sen*** | *entN* | *sen- other than RF122* | enterotoxin N | entN_11 | BA000017.4[1955741:1955768:r] | BA000017.4[1955492:1955513] |
|  |  |  | *sen-consensus* |  | hp_entN_611 | AF156894.1[1203:1230] | AF156894.1[1458:1479:r] |
|  | ***seo*** | *entO* | *-* | enterotoxin O | entO_11 | BA000017.4[1958936:1958962:r] | BA000017.4[1958904:1958925] |
|  | ***seq*** | *entQ* | *-* | enterotoxin Q | hp_entQ_611, hp_entQ_612, | AF410775.1[13213:13242], AF410775.1[13437:13467] | AF410775.1[13267:13288:r], AF410775.1[13404:13423:r], AF410775.1[13551:13570:r] |
|  | ***ser*** | *entR* | *-* | enterotoxin R | hp_entR-118699_PM5 | AB075606.1[750:775] | AB075606.1[783:802:r] |
|  | ***“setB1”*** | *-* | *setB1* | staphylococcal exotoxin-like protein, second locus | hp_setB1-118754_PM5 | AC027137.8[43435:43463] | AC027137.8[43473:43491:r] |
|  |  |  | *setB1-MRSA252* |  | hp_setB1-118757_PM5 | BX571856.1[1186711:1186738:r] | AC027137.8[43473:43491:r] |
|  | ***“setB2”*** | *-* | *setB2* | staphylococcal exotoxin-like protein, second locus | hp_setB2-118753_PM5 | AC027137.8[44254:44281] | AC027137.8[44295:44314:r] |
|  |  |  | *setB2-MRSA252* |  | hp_setB2-118756_PM5 | BX571856.1[1185785:1185812:r] | BX571856.1[1185730:1185749] |
|  | ***“setB3”*** | *-* | *setB3* | staphylococcal exotoxin-like protein, second locus | hp_setB3-118752_PM5 | AC027137.8[45064:45091] | AC027137.8[45097:45115:r] |
|  | ***“setC”*** | *selX* | *-* | staphylococcal enterotoxin-like toxin X | hp_setC-118758_PM5 | AC074316.7[11309:11335:r] | AC074316.7[11267:11288] |
|  | ***seu / sey*** |  | *-* | Enterotoxin U and/or Y | hp_entU_611 | AF156894.1[263:292], | AF156894.1[318:338:r] |
|  | ***spa*** | *-* | *-* | Protein A | hp_proteinA-118721_PM5, hp_proteinA-118722_PM5 | AB050857.1[923:949], AB050857.1[1454:1482] | AB050857.1[972:989:r], AB050857.1[1494:1511:r] |
|  | ***splA*** | *-* | *-* | serin protease A | hp_splA-118762_PM5 | AF271715.1[1447:1472] | AF271715.1[1488:1506:r] |
|  | ***splB*** | *-* | *-* | serin protease B | hp_splB-118763_PM5 | AF271715.1[2367:2392] | AF271715.1[2403:2420:r] |
|  | ***splE*** | *-* | *-* | serin protease E | hp_splE_611 | AF271715.1[4880:4903] | AF271715.1[4921:4941:r] |
|  | ***ssl01*** | *set6, set16* | *ssl01-COL*  *ssl01-Mu50*  *ssl01-MW2*  *ssl01-MRSA252* | staphylococcal superantigen-like protein 1**:**  set6-COL (SACOL468):  probe 1_11+probe 1_12  set6-Mu50 (SAV0422):  probe 1_11+probe 4_11  set6-MW2 (MW0382):  probe 2_11+probe 2_12  SAR0422 (from strain MRSA 252):  probe 2_11+probe 1_12 | hp_set6_probe 1_11 | BA000017.4[467156:467183], BA000018.3[441398:441425], AC069311.6[16791:16818] | BA000017.4[467190:467210:r], BA000018.3[441432:441452:r], AC069311.6[16825:16845:r] |
|  |  |  |  |  | hp_set6_probe 1_12 | BA000017.4[467359:467382], BA000018.3[441601:441624], BX571856.1[453059:453082] | BA000017.4[467396:467414:r], BA000018.3[441638:441656:r], BA000033.2[429765:429783:r] |
|  |  |  |  |  | hp_set6_probe 2_11 | BA000033.2[429525:429550], BX571857.1[428186:428211], BX571856.1[452856:452881] | BA000033.2[429559:429578:r], BX571857.1[428220:428239:r], BX571856.1[452890:452909:r] |
|  |  |  |  |  | hp_set6_probe 2_12 | BA000033.2[429731:429757], BX571857.1[428392:428418] |  |
|  |  |  |  |  | hp_set6_probe 4_11 | AC069311.6[16994:17017], CP000046.1[470856:470879] |  |
|  |  |  | *ssl01-RF122* | staphylococcal superantigen-like protein 1 allele from strain RF122 (ST151) | hp_ssl01_611 | AJ938182.1[412226:412252], AJ938182.1[412017:412045] | AC069311.6[17031:17049:r], AJ938182.1[412058:412076:r] |
|  | ***ssl02*** | *set7, set17* | *ssl02* | staphylococcal superantigen-like protein 2 | hp_set7-118747_PM5 | AJ938182.1[412951:412977] | AJ938182.1[413015:413034:r] |
|  |  |  | *ssl02-MRSA252* |  | hp_set7-118748_PM5 | BX571856.1[453788:453814] | AJ938182.1[413015:413034:r] |
|  | ***ssl03*** | *set8, set18* | *ssl03* | staphylococcal superantigen-like protein 3 | hp_set8-118749_PM5 | AP009324.1[468963:468988] | AJ938182.1[413847:413865:r] |
|  |  |  | *ssl03-MRSA252* |  | hp_set8-118761_PM5, hp_set8-118759_PM5 | AF094826.1[362:388], BX571856.1[454824:454850] | BX571856.1[454869:454886:r], AP009324.1[471026:471045:r] |
|  |  |  | *ssl03-RF122* |  | hp_ssl03_611 | AJ938182.1[413800:413827] | AJ938182.1[413847:413865:r] |
|  | ***ssl04*** | *set9, set19* | *ssl04-COL* | staphylococcal superantigen-like protein 4 | hp_set9-118750_PM5, hp_set9-118751_PM5 | AP009324.1[470721:470750], AP009324.1[470981:471007] | AP009324.1[470798:470819:r], AP009324.1[471026:471045:r] |
|  |  |  | *ssl04-MRSA252* |  | hp_set9-118761_PM5, hp_set9-118760_PM5 | AF094826.1[362:388], BX571856.1[456040:456064] | BX571856.1[454869:454886:r], AF094826.1[412:430:r] |
|  | ***ssl05*** | *set3, set20* | *ssl05* | staphylococcal superantigen-like protein 5 | hp_ssl05_612, hp_set3-118736_PM5 | AP009324.1[471546:471573], AP009324.1[471906:471934] | AP009324.1[471949:471966:r], AJ938182.1[415181:415202:r], AP009351.1[440788:440810:r] |
|  |  |  | *ssl05-MRSA252* |  | hp_set3-118737_PM5 | AF094826.1[936:962] | AF094826.1[972:993:r] |
|  |  |  | *ssl05-RF122* |  | hp_ssl05_611 | AJ938182.1[415143:415170] | AJ938182.1[415181:415202:r] |
|  | ***ssl06*** | *set21* | *ssl06-MW2* | staphylococcal superantigen-like protein 6 | hp_ssl06_611, hp_set21-118731_PM5 | AP009351.1[441864:441893], AP009351.1[441980:442007] | BA000033.2[435244:435264:r], AP009351.1[441902:441923:r] |
|  | ***ssl07*** | *set1, set22* | *ssl07* | staphylococcal superantigen-like protein 7 | hp_set1-118730_PM5 | AF188837.1[165:194] | AF094826.1[2235:2252:r] |
|  |  |  | *ssl07-FRI326* |  | hp_set1-118729_PM5 | AF188836.1[165:194] | AF094826.1[2235:2252:r] |
|  |  |  | *ssl07-MRSA252* |  | hp_set1-118728_PM5 | AF094826.1[2195:2224] | AF094826.1[2235:2252:r] |
|  | ***ssl08*** | *set12, set23* | *ssl08* | staphylococcal superantigen-like protein 8 | hp_set12-118727_PM5, hp_ssl08_611 | AP009324.1[473840:473867], AJ938182.1[417435:417464] | AP009324.1[473870:473890:r], AJ938182.1[417465:417488:r] |
|  | ***ssl09*** | *set5, set24* | *ssl09* | staphylococcal superantigen-like protein 9 | hp_ssl09_611, hp_set5-118740_PM5 | AJ938182.1[418433:418459], AP009324.1[474839:474865] | AJ938182.1[418479:418499:r] |
|  |  |  | *ssl09-MRSA252* |  | hp_set5-118741_PM5 | AF094826.1[3155:3181] | AF094826.1[3207:3228:r] |
|  | ***ssl10*** | *set4, set25* | *ssl10* | staphylococcal superantigen-like protein 10 | hp_ssl10_611, hp_set4-118738_PM5 | AJ938182.1[419736:419765], AP009324.1[476142:476170] | AJ938182.1[419782:419799:r] |
|  |  |  | *ssl10-MRSA252* |  | hp_set4-118739_PM5 | AF094826.1[4455:4481] | AF094826.1[4492:4509:r] |
|  | ***ssl11*** | *set2, set26* | *ssl11-COL* | staphylococcal superantigen-like protein 11 | hp_set2-118735_PM5 | AP009351.1[450382:450410] | AP009351.1[450419:450437:r] |
|  |  |  | *ssl11-MRSA252* |  | hp_set2-118733_PM5 | BX571856.1[464936:464964] | BX571856.1[464979:464999:r] |
|  |  |  | *ssl11-MW2* |  | hp_set2-118732_PM5 | AJ938182.1[423802:423830] | AJ938182.1[423871:423893:r] |
|  |  |  | *ssl11-Mu50* |  | hp_set2-118734_PM5 | AP009324.1[480162:480191] | AP009324.1[480193:480213:r] |
|  | ***sspA*** | *-* | *sspA-C66* | glutamylendopeptidase | hp_sspA_613, hp_sspA_611 | AF309515.1[1298:1323], AF309515.1[591:618] | AF309515.1[632:650:r], AF309515.1[1327:1347:r] |
|  |  |  | *sspA-MRSA252* |  | hp_sspA_613, hp_sspA_612, hp_sspA_611 | AF309515.1[1298:1323], BX571856.1[1064056:1064082:r], AF309515.1[591:618] | AF309515.1[632:650:r], AF309515.1[1327:1347:r] |
|  |  |  | *sspA-RF122* |  | hp_sspA_611, hp_sspA_614 | AF309515.1[591:618], AJ938182.1[991112:991137:r] | AF309515.1[632:650:r], AF309515.1[1327:1347:r] |
|  | ***sspB*** | *-* | *-* | staphopain B, protease | hp_sspB_611, hp_sspB_612 | AF309515.1[1546:1571], AF309515.1[2231:2260] | AF309515.1[1583:1603:r], AF309515.1[2264:2282:r] |
|  | ***sspP*** | *-* | *-* | staphopain A (staphylopain A), protease | hp_sspP_612, hp_sspP_611 | AC090969.3[16450:16478], AC090969.3[15944:15970] | AC090969.3[16483:16504:r], AC090969.3[15980:15998:r] |
|  | ***tet*(K)** | *tetK* | *-* | tetracycline resistance | hp_tetK-59251_PM1, hp_tetK-59250_PM1 | AB037671.1[32853:32881:r], AB037671.1[32936:32964:r] | AB037671.1[32784:32806] |
|  | ***tet*(M)** | *tetM* | *-* | tetracycline resistance | hp_tetM-59246_PM1 | AB039845.1[1410:1436] | AE014233.1[19842:19860] |
|  | ***tst1*** | *-* | *tst1_other than RF122* | toxic shock syndrome toxin 1 | hp_tst-59257_PM1 | AB084255.1[434:458] | AB084255.1[489:509:r] |
|  |  |  | *tst1_consensus* |  | hp_tst_611 | AB084255.1[595:623] | AB084255.1[630:649:r] |
|  | ***ugpQ*** | *-* | *-* | glycerophosphoryl diester phosphodiesterase, associated with *mecA* | hp_ugpQ_611 | AB033763.2[34349:34374:r] | AB033763.2[34320:34338] |
|  | ***vanA*** | *-* | *-* | vancomycin resistance gene | 18,2-vanA | AB247327.1[16135:16162] | AB247327.1[16238:16257:r] |
|  | ***vanB*** | *-* | *-* | vancomycin resistance gene from enterococci and *Clostridium* | 19,3-vanB  vanB_11 | AE016830.1[2213146:2213171:r], AE016830.1[2213149:2213173:r] | AE016830.1[2213051:2213068] |
|  | ***vanZ*** | *-* | *-* | teicoplanin resistance gene from enterococci | 20,3-vanZ | AB247327.1[19318:19343] | AB247327.1[19367:19386:r] |
|  | ***vatA*** | *-* | *-* | virginiamycin A acetyltransferase | vatA_15,3 | AF117258.1[2296:2323:r] | AF117258.1[2229:2247] |
|  | ***vatB*** | *-* | *-* | acetyltransferase inactivating streptogramin A | vatB_16,3 | U19459.1[543:570] | U19459.1[659:680:r] |
|  | ***vga*** | *-* | *vga* | ATP binding protein, streptogramin-A-resistance | vga_17,3 | AF117259.1[3729:3756] | AF117259.1[3925:3944:r], AF117259.1[3779:3799:r] |
|  |  |  | *vga-BM 3327* |  | vgaA_18,3 | AF186237.2[6470:6497] | AF186237.2[6559:6578:r] |
|  | ***vgb*** | *-* | *-* | virginiamycin B hydrolase | vgb_19,2 | AF117258.1[3342:3369:r] | AF117258.1[3261:3283] |
|  | ***vraS*** | *-* | *-* | sensor protein | hp_vraS_612 | AB035448.1[4748:4773] | AB035448.1[4783:4802:r] |
|  | ***vwb*** | *-* | *vwb-COL* | van Willebrand factor binding protein | hp_vwb_612, hp_vwb_615 | AC074317.5[3364:3392:r], AB240058.1[1171:1196] | AC074317.5[2314:2333], AC074317.5[3329:3351] |
|  |  | *-* | *vwb-MRSA252* |  | hp_vwb_613, hp_vwb_615 | BX571856.1[891899:891927], AB240058.1[1171:1196] | BX571856.1[891952:891972:r], AC074317.5[2314:2333] |
|  |  | *-* | *vwb-Mu50* |  | hp_vwb_614, hp_vwb_615 | AB240058.1[730:758], AB240058.1[1171:1196] | AB240058.1[771:791:r], AB240058.1[1204:1223:r] |
|  |  | *-* | *vwb-RF122* |  | hp_vwb_611, hp_vwb_615 | AJ938182.1[821019:821046], AB240058.1[1171:1196] | AC074317.5[2314:2333], AJ938182.1[821064:821083:r] |
|  | ***xylR*** | *mecR2* | *-* | homolog of xylose repressor, associated with SCC*mec*-elements | hp_xylR_611 | AB037671.1[21440:21468:r] | AB037671.1[21410:21429] |
